# MEGA PROTAC, MEGA DOCK-based PROTAC mediated ternary complex formation pipeline with sequential filtering and rank aggregation

**DOI:** 10.1038/s41598-024-83558-2

**Published:** 2025-02-14

**Authors:** Sadettin Y. Ugurlu, David McDonald, Ramazan Enisoglu, Zexuan Zhu, Shan He

**Affiliations:** 1 School of Computer Science, University of Birmingham, Edgbaston, Birmingham, B15 2TT UK; 2 AIA Insights Ltd, Birmingham, UK; 3 School of Science and Technology, City St George’s, University of London, Northampton Square, London, EC1V 0HB UK; 4 National Engineering Laboratory for Big Data System Computing Technology, Shenzhen University, Shenzhen, China

**Keywords:** PROTAC, Sequential filtration, Rank aggregation, Docking, Mediated ternary complex, Proteolysis-targeting chimaeras, Computational biology and bioinformatics, Drug discovery

## Abstract

Proteolysis-targeting chimaeras (PROTACs), which induce proteolysis by recruiting an E3 ligase to dock into a target protein, are acquiring popularity as a novel pharmacological modality because of the unique features of PROTAC, including high potency, low dosage, and effective on undruggable targets. While PROTACs are promising prospects as chemical probes and therapeutic agents, their discovery usually necessitates the synthesis of numerous analogues to explore variations on the chemical linker structure exhaustively. Without extensive trial and error, it is unknown how to link the two protein-recruiting moieties to facilitate the formation of a productive ternary complex. Although molecular docking-based and optimization pipelines have been designed to predict ternary complexes, guiding rational PROTAC design, they have suffered from limited predictive performance in the quality of the ternary structure and their ranks. Here, MEGA PROTAC has been designed to enhance the performance in quality and ranking of ternary structures. MEGA PROTAC employs MEGADOCK to execute docking for protein-protein complexes (PPCs). The docking establishes an initial exploration area for PPCs. A sequential filtration strategy combined with rank aggregation is employed to choose a subset of PPCs for grid search. Once candidate PPCs are selected, a grid search method is used separately for translation and rotation. The remaining proteins have been grouped into clusters, and MEGA PROTAC further filters these clusters based on the energy score of the proteins within each cluster. MEGA PROTAC utilises rank aggregation to choose the best clusters and then employs MEGADOCK to dock PROTAC into the selected PPCs, forming a ternary structure. Finally, MEGA PROTAC was tested on 22 cases to compare with the state-of-the-art method, Bayesian optimisation for ternary complex prediction (BOTCP). MEGA PROTAC outperformed BOTCP on 16 test cases out of 22 cases, achieving a higher maximum DockQ score with an **18% higher mean** and **35% higher median**. Also, MEGA PROTAC exhibited **75% superior ranks** and a reduced cluster number for maximum DockQ score compared to BOTCP. Also, MEGA PROTAC outperforms BOTCP by achieving a **twofold improvement** in locating the first acceptable DockQ scores, with a more significant proportion of near-native structures within the detected cluster.

## Introduction

Cellular functions, including proliferation, differentiation, and cell mortality, are contingent upon cellular protein degradation. Ubiquitin-dependent degradation is one of the most essential post-translational pathways for protein regulation^1^. Ubiquitin-dependent degradation is a nascent treatment technique that promotes the destruction of the target protein instead of merely blocking its function^2,3^. The approach utilises monofunctional degraders, commonly referred to as molecular glues or heterobifunctional degraders, such as Proteolysis Targeting Chimeras (PROTACs), which function as proximity-inducing compounds^1^. PROTACs selectively degrade a targeted protein using the ubiquitin-dependent degradation system^4^. PROTACs are heterobifunctional molecules consisting of two ligands. One ligand, called the “anchor,” is responsible for binding to an E3 ubiquitin ligase. The other ligand, known as the “warhead,” binds to a specific protein of interest. A chemical linker joins these two ligands to construct PROTACs^5^. The substrate binding domain (SBD) of an E3 ubiquitin (Ub) ligase is bound by an “anchor” ligand, whereas a specific protein of interest (POI) to be targeted is bound by a “warhead” ligand. By interacting with proteins within cells, the PROTAC facilitates the recruitment of the POI to a ternary complex (TC) alongside the E3 ligase^6^. The E3 ligase is in a complex with an activated E2 ligase that is loaded with ubiquitin. Establishing the ternary complex brings the entire ensemble into close proximity with the protein of interest. This process results in the (poly)-ubiquitination of the POI at specific lysine residues, thereby designating it for degradation through the action of the 26S proteasome^7–9^.

Traditional small molecule inhibitors of proteins function by inhibiting protein function, whereas protein-targeted degraders, such as PROTACs, function by degrading proteins via the proteasome, resulting in distinct biological properties, better selectivity, and potency^10^. In particular, high selectivity and potency reduce the dose levels and toxicity for disease treatment, including complex diseases like cancer^11–13^. Furthermore, unlike conventional pharmaceuticals, which require strong binding, PROTACs can induce protein degradation with even a weak binding^4,14^. A single PROTAC, having oral bioavailability^15^, can substoichiometrically act to degrade several instances of the target protein^14–17^. Additionally, PROTACs offer a range of benefits that address the existing constraints associated with conventional medicines, including “undruggable targets”, poor therapeutic effect, short duration of action, and drug resistance^4,15–18^. For instance, STAT3, a transcription factor essential for cell proliferation and death, has been deemed immune to manipulation by using small molecule inhibitors. Bai et al. created SD-36, a potent and selective STAT3 small-molecule degrader, in 2019, which completely and permanently regressed tumours in xenograft mouse models^19^.

The technical challenge of designing three constituent elements for constructing a PROTAC, which would yield a pharmacological effect with desirable drug-like qualities, arises from the intricate interplay and coherence required among the many components of PROTACs^4^. One additional problem lies in efficiently and proficiently conducting screening processes to identify target protein ligands suitable for utilisation in PROTACs, focusing on those that target protein–protein interactions^13^. The human genome is responsible for encoding many E3 ubiquitin ligases, exceeding 600 in total. However, the utilisation of E3 ligases “anchor” in the design of PROTACs is limited to a small number, namely VHL, CRBN, cIAPs, and MDM2^13^. In addition to the extensive search space associated with E3 ligases, exploring linkers and warheads considerably expands the search region for PROTACs since the three of them should be screened simultaneously. Therefore, the method should be considerably faster for screening such an extensive search space. Also, research should be made to develop expeditious and precise methodologies for constructing ternary complexes to comprehend the underlying mechanisms of PROTACs and surmount their inherent design constraints.

There is a scarcity of research in the existing literature regarding enhancing performance in ternary structure in-silico construction due to the highly time-consuming and expensive nature of web-lab-based screening to validate the in-silico models. In one of the initial investigations, ML Drummond et al. employed protein–protein docking to explore protein–protein complexes and conducted a shape search for PROTAC^4^. Also, RosettaC employed the PROTAC conformation space in order to sample ternary structures^16^. The ternary structure was clustered, and then, the ternary structure was determined using the Rosetta score^16^. Bai et al. employed a scoring approach that combined geometric and energetic considerations, in addition to utilising the RosettaDock score, in order to enhance performance^17^. To enhance the performance of ternary structure creation, Weng et al. employed refinement techniques based on FRODOCK and RosettaDock^4^. Furthermore, Weng et al. employed a re-scoring and grouping approach to ascertain the ultimate ranking of ternary structures^4^. In recent studies^3,20,21^, both the sampling and rescoring methodologies to define ternary structure have been improved by using energy-based filtration and VoroMQA-based ranking. State-of-the-art techniques, including Bayesian optimisation for ternary complex prediction (BOTCP)^14^, enhanced the performance of PROTAC screening. BOTCP is a machine learning strategy for predicting PROTAC-mediated ternary complex formations^14^. A fitness score combines estimates of protein–protein interactions and PROTAC conformation energy calculations^14^. This makes it possible to find candidate ternary structures using samples^14^. BOTCP introduces innovative scores for the purpose of filtering and reranking. These scores are the stability of PROTAC (measured using the Autodock-Vina-based PROTAC stability score) and the constraints imposed by protein interactions (measured using the TCP-AIR score)^14^. There is still potential for further improvement in the performance of PROTAC screening, especially in pre-refinement steps. The pre-refinement steps of recent studies suffered from limited performance because of limited filtration approaches and ranking performance.

A novel in-silico ternary structure prediction pipeline, MEGA PROTAC, has been developed to improve the quality of ternary structures and ranking performance. The primary docking program selected is MEGADOCK^22^ due to its remarkable speed and ability to generate diverse outputs due to its distinctive parameters, including penalty scores for both target and ligand proteins. MEGADOCK is employed for protein–protein docking to create an initial search space for ternary structures. The initial structures have been cleaned out using sequential filtration, and the top potential ones have been selected using rank aggregation. The protocol utilises a grid search to explore different axes and rotations on Euler degrees to precisely locate potential structures by increasing the search region. Increasing the size of the search area significantly improves the likelihood of identifying a “True” ternary structure within it. Therefore, the same sequential filtration approach integrated with rank aggregation has been used to select the most potential structures. Then, the remaining potential structures were clustered, and clusters were filtered out based on whether a protein with low energy scores existed. Finally, MEGA PROTAC docks PROTAC into potential structures using MEGADOCK. The docking produces ternary structures for the PROTAC. MEGA PROTAC is freely and publicly available for academic use: https://github.com/yauz3/MEGA-PROTAC

## Materials and methods

MEGA PROTAC was evaluated against advanced techniques using all experimentally valid available 3D structures of 22 ternary structures published in BOTPC^14^ (For data and code, click Here.). The evaluation included standard performance evaluation measures such as DockQ score, fnot, and RMSDs. The remainder of this section is divided into four primary subsections: (i) Comprehensive overview of the complete MEGA PROTAC pipeline, (ii) Comparison with state-of-the-art methods, (iii) Preparation of test sets, and (iv) Performance evaluation.

### Comprehensive overview of the complete MEGA PROTAC pipeline

**Fig. 1:**
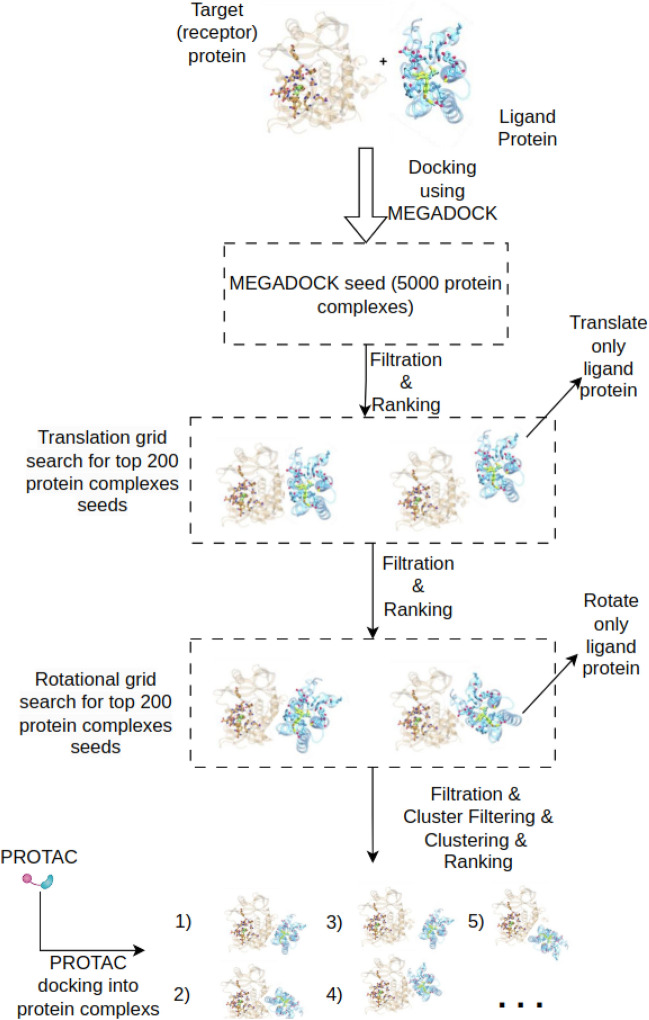
The diagram depicts the step-by-step process of the MEGA PROTAC methodology. (i) Preparation of input files includes the preparation of target and ligand proteins that have their ligands. (ii) Protein–protein docking is performed to have an initial search area as pre-grid refinement candidate PPCs. 5000 PPCs were produced using MEGADOCK. (iii) Filtration of PPCs is to remove unpromising structures based on filtration criteria. (iv) Rank aggregation and Grid searches cover selecting the subset (200) of unfiltered PPCs by using rank aggregation, then using them in grid search to improve the quality of PPCs. (v) Clustering is to classify unfiltered PPCs. (vi) Clustering Filtration filters unpromising clusters based on energy score. (vii) Ranking for reclustering uses rank aggregation for re-clustered PPCs. (viii) Finally, PROTAC is docked into PPCs using MEGADOCK.

The MEGA PROTAC procedure comprises a series of phases aimed at enhancing the quality of the ternary structures and their ranking to improve practical usage. The MEGA PROTAC method consists of eight stages with default parameters that can be adjusted using the scripts provided in the GitHub repository. The eight stages are : (i) Preparation of Input Files, (ii) Protein–protein docking involving their ligands, (iii) Filtration of protein complexes, (iv) Rank aggregation and Grid search for local optimisation of PPI complexes, (v) Clustering of Filtered Grid Search Complexes, (vi) Cluster Filtration, (vii) Re-clustering PPCs after cluster filtration, (viii) Ranking for reclustered PPCs and (ix) PROTAC docking into PPCs (Fig. 1).

#### Preparation of input files

MEGA PROTAC requires the E3+anchor and POI+warhead complexes in .pdb format for docking purposes, as shown by previous studies, such as BOTCP^14^, and other studies^4,16^. The files must be free of water molecules and deprotonated by eliminating hydrogens. Furthermore, MEGA PROTAC requires PROTAC structure in a .pdb file that can be used for PROTAC docking.

#### Protein–protein docking involving their ligands

While Weng et al.^4^ utilised the FRODOCK software, a standard protein–protein docking program, for the purpose of protein docking, the primary conventional protein–protein docking program utilised in our pipeline is MEGADOCK. This was chosen for its advantages, including rapidity in screening more PROTAC possibilities and unique parameters (Table 1) to optimise MEGADOCK output by following an evaluation of the molecular docking programs in the existing literature^22,23^.

**Table 1:** The summary feature and groups of molecular docking programs in the literature.

Docking program	Docking type	Input type	Ternary docking	Features
Vina	Local docking	Small molecule-protein	No	High performance: 81% accuracy^24^
				Ease of use
				Common
				A high number of different pose locations
PLANTS	Local docking	Small molecule-protein	No	High performance: 87%^25,26^
				Relatively fast
				A high number of different pose locations
GalaxyDock3	Local docking	Small molecule-protein	No	High performance^27^
				Ease of use
				A high number of different pose locations
				Full Ligand Conformational Flexibility^27^
Rosetta	Local docking	Protein–protein	**Yes**	High performance^28,29^
				The high number of independent structures^45,29^
				Moderate or low speed^30^
FRODOCK 2.0	Local docking	Protein–protein	**Yes**	Extra knowledge-based potential^25^
				High Performance^25,31^
CoBDock	Global (blind) docking	Small molecule-protein	**No**	High performance^32^
				High automation^32^
MEGADOCK 4.0	Global (blind) docking	Protein–protein	**Yes**	High speed^22,23^
				Relatively high-performance^43,29^
ZDOCK	Global (blind) docking	Peptide–protein, protein–protein	**Yes**	High performance: 85.71%^33^
				Blind (Global) docking
				A high number of poses in similar locations
LightDock	Global (blind) docking	Protein–protein, peptide-protein, DNA-protein	No	Conformational flexibility^34^
				A variety of scoring functions^44,29^

The categorisation of molecular docking can be based on two factors: the type of docking and the type of input. Docking types can be classified into two categories: (i) local docking and (ii) global (blind) docking. The process of local docking involves the execution of docking algorithms on a designated and predetermined position of the target protein. In cases where the binding site’s precise position is unknown, it becomes necessary for molecular docking systems to do a comprehensive search of the complete protein structure to identify potential binding sites and subsequently execute the docking process. Such a process is called global (blind) docking. Additionally, it is possible to categorise molecular docking programs into three distinct classes according to their inputs: (i) small molecule-protein docking programs, (ii) peptide-protein docking programs, (iii) protein–protein, and (iv) Nucleic acid-protein docking programs. The molecular docking programs have undergone testing to determine their capability to execute ternary docking. Ternary docking represents the ability to perform docking for two proteins and one ligand. Finally, the table demonstrates the features of docking programs in the literature.

*Selection of molecular docking program* To determine the primary molecular docking method for this study, Four molecular docking algorithms known for their ternary docking capabilities were examined: ZDOCK^33^, MEGADOCK 4.0^22^, FRODOCK^31^, and RosettaDock^28^. Despite the utilisation of RosettaDock and FRODOCK in contemporary procedures^4,16,17^, they nevertheless entail certain drawbacks. Weng et al. employed RosettaDock to enhance the efficacy of FRODOCK^4^, so suggesting that RosettaDock exhibits superior accuracy compared to FRODOCK. However, the RosettaDock approach is deemed to be time-inefficient due to the requirement of several hours to complete^30^. In contrast, on average, the ZDOCK and FRODOCK take several minutes to accomplish protein–protein docking^35,36^. Fortunately, MEGADOCK can complete docking procedures in a matter of seconds^22,23^. According to the preceding debate, it has been determined that MEGADOCK demonstrates a notably superior velocity in comparison to FRODOCK and ZDOCK, resulting in a speed augmentation of up to 60-fold^22,23,35,36^. Besides MEGADOCK’s fast process ability, it is user-friendly and provides unique parameters, including a core penalty score to control the diversity of outputs^22^. Thus, MEGADOCK has been chosen as the primary docking program for the MEGA PROTAC.

*Performing protein–protein docking* Similar to prior research on the development of PROTAC ternary structures^4,14^, MEGA PROTAC conducts docking utilising the E3+anchor and POI+warhead in the .pdb file format, utilising MEGADOCK. To optimize the MEGADOCK pre-grid refinement candidate PPCs, a randomly selected individual structure (6HAY-BA) is employed, as done in prior experiments^14,16^. The parameters (Supplementary Information) of MEGADOCK have been optimised by visual examination using Pymol^37^ to achieve a wide range of ligand-protein locations around the target protein. When the majority of ligand-protein locations differ from each other, one of them likely represents an appropriate site. Consequently, 5000 temporary “ternary structures” were generated for each input without including a linker. These 5000 complexes serve as a pre-grid refinement candidate PPC for initiating the grid search following the application of filters.

#### Filtration of protein complexes

The process of filtering protein complexes that have potential leads to improved quality of structures and their ranking. Prior research, such as PRosettaC^16^, employs filtration techniques, such as ligand distance-based filtration, to remove protein complexes. For MEGA PROTAC, two types of filtration were devised: (i) rough ligand-based filtration and (ii) protein-based filtration.

*Rough ligand-based filtration* Rough ligand-based filtration, as a filtration approach based on the distance between anchor and warhead, is the fastest technique employed in MEGA PROTAC to eliminate unfavourable positions and orientations of ligand-protein on a target protein. Thus, many unpromising protein complexes can be rapidly filtered in seconds, whereas protein-based filtering takes minutes to hours. Rough ligand-based filtration is advantageous over protein-based filtration, primarily targeting protein interfaces while disregarding the warhead and anchor’s placement. Thus, To optimize time and computational resources and address the limitations of protein-based filtration, a preliminary rough ligand-based filtration step was implemented at the beginning of the filtration process by following PRosettaC^16^.

Following the PRosettaC study^16^, the technique of ligand-based filtering was employed to remove PPCs that showed limited potential. In the PRosettaC^16^ study, the minimum and maximum distances observed between the anchor and warhead ranged from 8 to 15 Å. In order to mitigate the risk of losing potential protein complexes, a range of 3-20 Å, representing the minimum and maximum distances between the anchor and warhead, was employed. The calculation utilised the Euclidean distance between the mass centre of the anchor and the warhead. PPCs with distances of 3 Å or 20 Å have been kept for protein-based filtering since, while ligand-based filtration is effective in removing unpromising protein complexes with an anchor and warhead oriented towards opposite sides, more sophisticated protein-based filtration methods are needed to eliminate deceiving protein complexes.

**Fig. 2:**
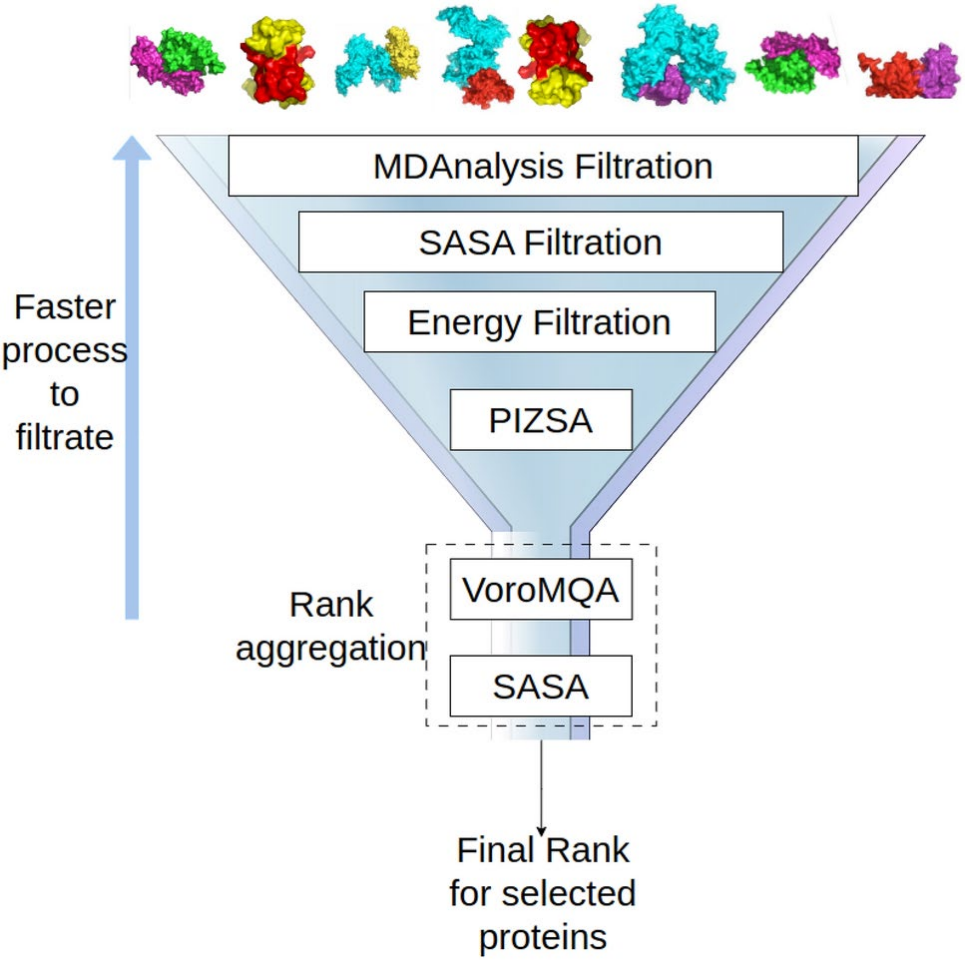
The graphic illustrates protein-based filtrations performed using MDAnalysis, SASA, Energy, and PIZSA. The filtrations are performed in a prioritised sequence, starting with faster methods and progressing to slower ones, in order to maximise computational efficiency. After the process of filtration, MEGA PROTAC utilises rank aggregation of VoroMQA and SASA scores to determine the most favourable proteins. Proteins with bigger SASA values are given higher priority while assessing the quality of PPCs using VoroMQA. This is because larger SASA values suggest wider gaps between proteins, making it easier for PROTAC molecules to bind optimally. MEGA PROTAC utilised VoroMQA to closely monitor and maintain good quality by examining larger spaces between proteins. Consequently, rank aggregation identifies the PPC with the highest quality and more significant gaps, which is suitable for PROTAC to fit.

*Protein-based filtration* A protein-based filtration method was used to eliminate protein complexes that exhibited limited potential. Sequential filtration offers the additional benefit of time efficiency. As an illustration, rough ligand-based filtration has been identified as the most expeditious filtration technique in the realm of research, capable of efficiently eliminating numerous proteins within minutes. Similarly, the faster protein-based filtration approach is positioned at the onset of protein-based filtration to optimise efficiency during extensive screening processes. Consequently, MEGA PROTAC employs (i) MDAnalysis score, stability-based filtration, (i) SASA-based filtration, (ii) Energy-based filtration, (iv) Protein Interaction Z-score quality-based filtration and (v) VoroMQA-based quality assessment for rank aggregation (Fig. 2) (Details about protein filtration can be found in Supplementary Information). Before submitting PPCs to the sequential filters (Fig. 2), PyMol^37^ was employed to remove the anchor and warhead.

*Summary of sequential filtration*: During the ubiquitination process, the three-dimensional structure (ternary structure) of the target protein plays a crucial role. The stability of the ternary structure ensures the protein maintains its proper conformation under cellular conditions. Unstable structures are often targeted for degradation before they can exert any pharmacological effect. Tools like MDAnalysis, SASA, and energy analysis can be employed after rough ligand-based filtration to evaluate the stability of protein complexes involved in ubiquitination. These methods assess stability factors, such as atomic fluctuations and potential energy. Also, a stable protein structure is a strong indicator of high quality. High-quality proteins are well-suited for their designated functions, performing them efficiently and safely. Therefore, VoroMQA is often used to assess the overall quality of protein complexes. Also, while PIZSA focuses on the strength of protein interaction, combining this data with other methods like MDAnalysis, SASA, energy analysis, and VoroMQA is important to assess PPCs. Such a comprehensive filtration approach provides a more complete picture of protein quality, stability, and the biological relevance of the protein–protein interaction. Sequential filtration was integrated with rank aggregation to increase the performance and robustness of MEGA PROTAC.

#### Rank aggregation and grid search for local optimisation of PPI complexes

During each stage of the MEGA PROTAC process, candidates are filtered according to the criteria described above. They are then ranked using our rank aggregation approach to identify the most promising structures. Rank aggregation combines many rankings of the same items into a single ranking representing a consensus (More details can be found in Supplementary Information). It helps to reduce the inherent bias seen in individual ranks, resulting in a more reliable ranking system. Moreover, it allows you to incorporate rankings from many sources that may use different grading systems.

*Rank aggregation* The describing distance between proteins poses a significant challenge in selecting “True” protein complexes. In order to facilitate the docking of PROTAC between proteins, it is necessary to increase the distance between them beyond the typical range so that such a large ligand, PROTAC, can fit into that big space between proteins. In our pipeline, SASA is the most suitable value to represent such an unusual space between proteins. A higher SASA value indicates a more accessible surface on the protein–protein complexes. A higher accessible surface can be obtained when proteins are located far away from each other. Therefore, SASA has been selected as a component of our rank aggregation.

High-quality proteins are well adapted for their intended tasks, efficiently executing them and ensuring safety. A protein structure that remains stable is a reliable indicator of excellent quality. In other words, protein quality encompasses protein stability, making the protein score a more comprehensive measure than stability alone. Thus, the quality assessor, VoroMQA, can provide more valuable data than other filtering components, making it suitable for assessing protein stability. Consequently, VoroMQA has now become the second component in our rank aggregation.

In summary, the total SASA area and VoroMQA quality score were employed in rank aggregation to identify the protein complex with the highest qualification while maintaining a significant space between proteins. In that space, PROTAC can easily fit to construct a ternary structure.

*Translational grid search* In the literature, Fast Fourier transforms (FFTs)-based rigid body docking is the standard method for protein docking^38,39^. FFT is a technique for thoroughly examining all possible rigid body orientations of a protein receptor and a ligand conformer within the discretised conformational space. During the sampling procedure, the protein remains stationary while the ligand undergoes rotational and translational movements^40^. The translations are sampled on a 3D grid, whose size can range from 1.0 to 6.0 Angstrom spacing^40–43^.

**Fig. 3:**
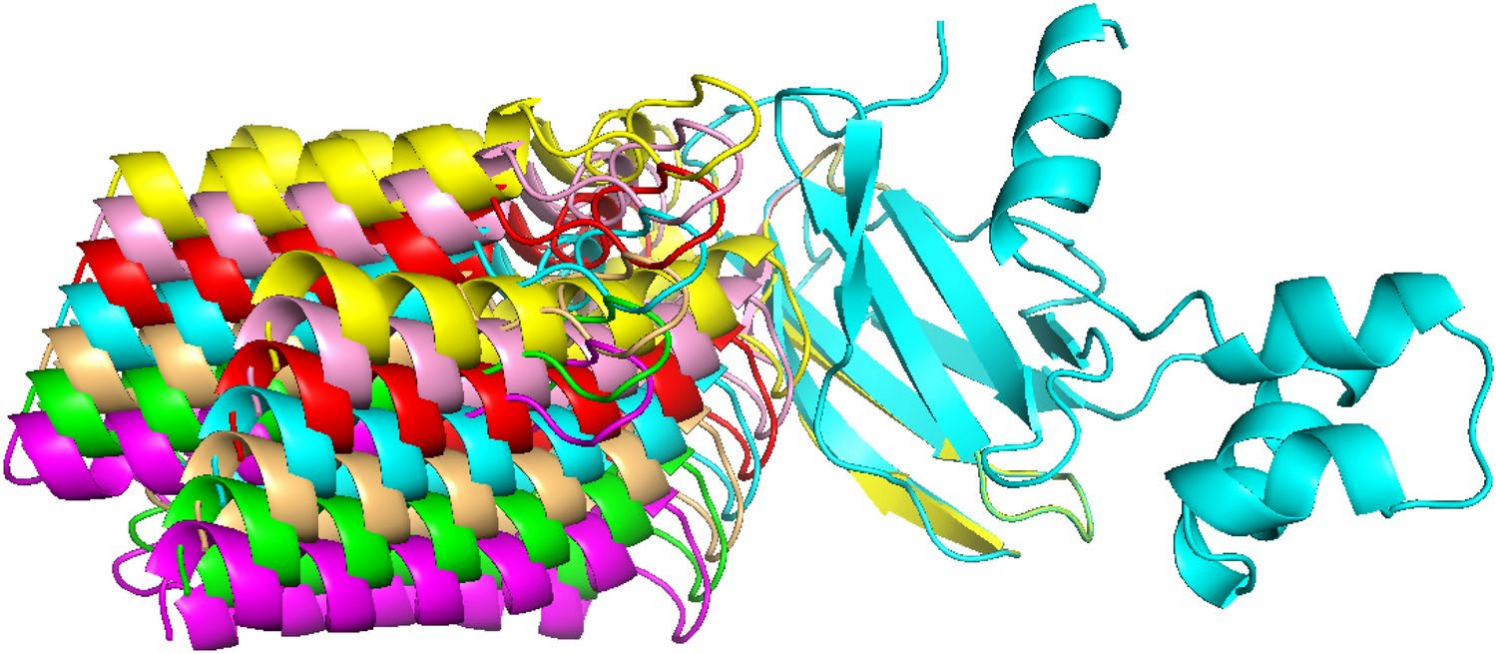
The figure represents the rotating grid search conducted on the 6HAY-BA protein, wherein the MEGA PROTAC rotated the ligand protein. The cyan colour represents translational pre-grid refinement candidate PPCs; hence, the original coordinates were set to 0,0,0 for translational structure. The colours red, pink, and yellow sequentially exhibit Euclidean distances of 1.5, 3, and 4.5 Å on all axes. The colours, including light orange, green, and magenta, also have Euclidean distances of -1.5, -3, and -4.5 Å on all axes, respectively.

The research conducted by PRosettaC^16^ demonstrated that the most favourable spacing between anchor and warhead is 12 Angstroms (Å). Thus, a lower grid spacing of 1.5 Å has been selected to optimise ternary structures by following Hou et al.^43^. As a result, PyMol^37^ has been employed to iteratively translate the ligand-protein across a grid with a resolution of 1.5 Å, ranging from +-4.5 Å in all three axes from the initial location of the protein (Fig. 3), where MEGA DOCK predict.

The filtering method described in section 2.1.3 was rigorously followed, ensuring each step was precisely executed. Methodological consistency and rigour were ensured in our work by faithfully implementing each stage of the filtering procedure. Consequently, the most promising top 200 translated PPCs have been selected for a rotational grid search.

*Rotational grid search* Fast Fourier transforms (FFT) techniques involve scanning translational space using different discrete orientations of one protein relative to the other to find the best geometric complementarity^38,39,44^. At the same time, a grid size of 10$^\circ$ or 5$^\circ$ is typically utilised for rotational search^40,44^, a larger grid size can be employed to conserve computational resources and reduce time consumption. Using relatively large Euler angle rotation grids of approximately 15$^\circ$ to 30$^\circ$ degrees is sometimes required for a systematic docking simulation^45^. Performing a grid search with larger degrees, such as +-60$^\circ$, and a smaller grid size, like 5$^\circ$, significantly increases the time needed for the search. However, this approach has the potential to improve performance. Hence, it is crucial to carefully choose the upper bound and dimensions of the rotation grid search to achieve a balance between performance and time efficiency.

MEGA PROTAC aims to balance time efficiency and performance. The selection of the upper bound degree and grid size strongly impacts this balance. MEGA PROTAC benefits MEGADOCK by allowing it to search for and eliminate all degree possibilities. Also, MEGA PROTAC can improve those possibilities by performing a translational gird search. Also, once the angle between ligands, depending on the ligand-protein mass centre, exceeds 45$^\circ$, the distance between them can increase. At the beginning of our filtration approach, most of these complexes can be filtered. Therefore, MEGA PROTAC can balance time efficiency and performance by performing an extensive rotational search in an upper bond degree (15$^\circ$ to 30$^\circ$).

**Fig. 4:**
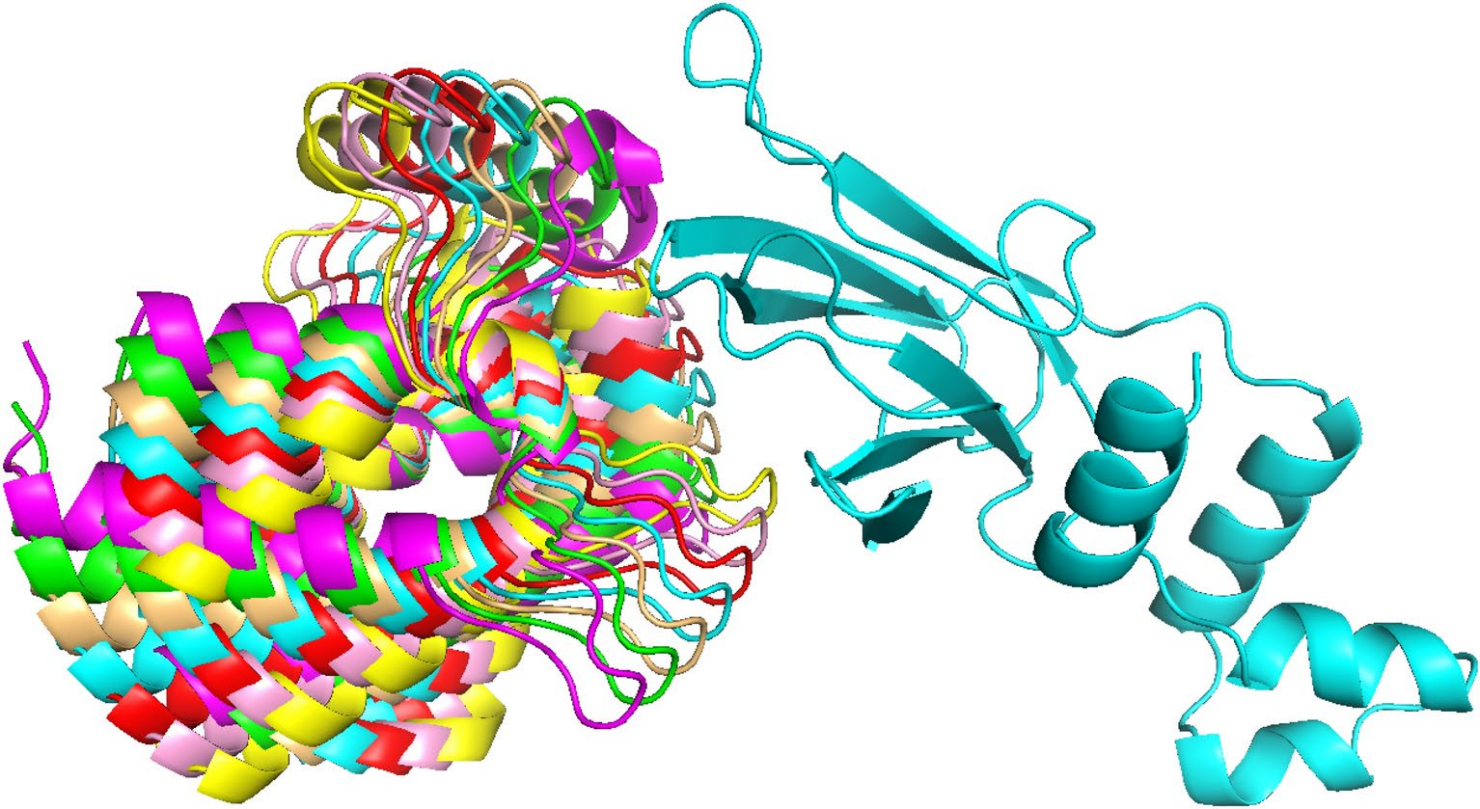
The figure represents the rotating grid search conducted on the 6HAY-BA protein, wherein the MEGA PROTAC rotated the ligand protein. The cyan colour represents the translational pre-grid refinement candidate PPCs; hence, the rotational angles were set to 0,0,0. The colours red, pink, and yellow exhibit Euclidean angles of 5, 10, and 15 on all axes. The colours, including light orange, green, and magenta, also represent angles of -5, -10, and -15 degrees on all axes.

MEGA PROTAC utilises a filtration method based on the distance between ligands to remove ligands that are significantly far from each other. Therefore, searching at a modest Euler angle of +-15$^\circ$ is likely sufficient for finding a more qualified protein structure while utilising a smaller grid size of 5$^\circ$. In addition, angles greater than 15$^\circ$ result in an exponential increase in the number of rotated proteins, necessitating substantial computational resources and time (Fig. 4). MEGA PROTAC default parameters have been selected to increase performance in an acceptable run time. These parameters also contribute to the practical usage of MEGA PROTAC thanks to enhanced performance in an acceptable run time.

In summary, the top 200 translated proteins determined using rank aggregation were utilised in the rotational grid search. Using PyMol, the translated protein was rotated through an angular grid of +-15 degrees using an angular resolution of 5 degrees. The resulting rotational proteins have been filtered according to our filtration criteria (described in section 2.1.3). Before constructing the ternary structure, the remaining proteins were retained for clustering and clustering filtration.

#### Clustering of filtered grid search complexes

Clustering outputs enhance practical usability by allowing users to quickly browse across distinct clusters instead of validating each individual ternary structure. Following previous studies^4,14^, the fraction of common contacts (FCCs) has been used to cluster promising outputs. FCCs are anticipated to save computational time significantly by eliminating the need for the structural alignment step. Therefore, the FCC approach has been used in MEGA PROTAC to cluster filtered grid search complexes.

Following the recent studies^14,20,21^, MEGA PROTAC categorises proteins into clusters by using the FCC method. According to recent studies^14,20,21^, the FCC method is employed to categorise proteins. The following FCC parameters were used: a similarity criterion of 0.5 and a minimum number of proteins in a cluster of 2. The clusters have been filtered out in the next step, cluster filtration.

#### Cluster filtration

**Fig. 5:**
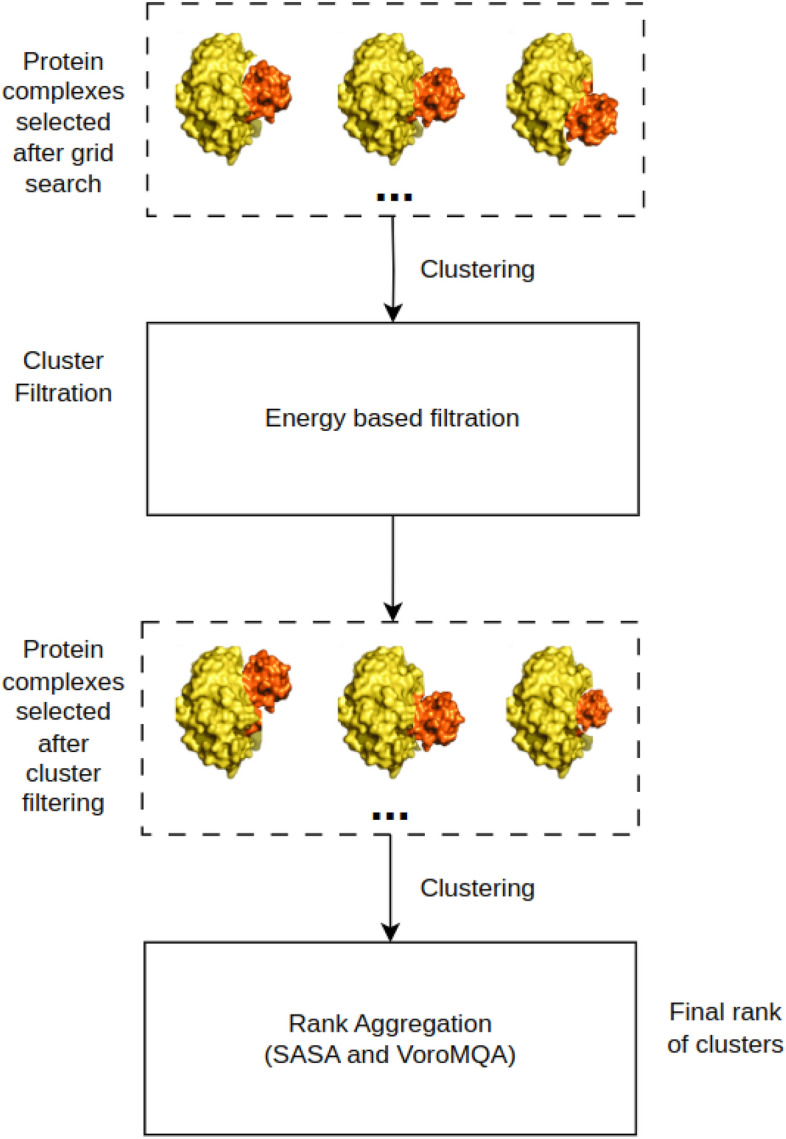
The figure demonstrates how to use rank aggregation applications in MEGA PROTAC. MEGA PROTAC uses MEGADOCK pre-grid refinement candidate PPC in its grid search to find promising 3D structures. These proteins are used to obtain clusters, and then MEGA PROTAC uses an energy-based filtration strategy to filtrate clusters using the UFF force field in OBenergy. Then, MEGA PROTAC reclusters the rest of the protein after cluster filtering, and finally, MEGA PROTAC performs clustering rank aggregation based on SASA and VoroMQA to obtain final ranks for clusters.

Following BOTCP^14^, MEGA PROTAC utilised clustering filters to remove unpromising clusters. To achieve robust and high-performance filtering, energy-based cluster filtering was employed, similar to the TCP-AIR score used in BOTCP^14^ (Fig. 5). Our UFF total energy has been used to save time and cost instead of TCP-AIR, which takes around 16 hours for a structure^46^.

Following BOTCP^14^, the same approach was applied to filter clusters in two steps: (i) Each protein has been ordered using its energies. (ii) the 25% of proteins with the lowest energy PPCs have been selected. Once a cluster has one of the selected PPCs with lower energy, the cluster is kept for further; otherwise, the cluster is eliminated. Finally, the proteins in selected clusters were re-clustered and ranked clusters, as BOTCP did^14^.

#### Re-clustering PPCs after cluster filtration

Identical parameters, defined in **Clustering of Filtered Grid Search Complexes**, have been employed to re-cluster PPCs. The FCC approach is utilised to classify proteins using a similarity criterion of 0.5 and a minimum protein number in a cluster count of 2. Non-clustered proteins have been eliminated.

#### Ranking for re-clustered PPCs

Ranking re-clustered PCCs significantly enhances the practicability of MEGA PROTAC. To ensure accuracy and consistency, the maximum SASA and VoroMQA protein values within the re-clustered PCCs were selected to represent their respective groups, following the same approach used in BOTCP^14^. By employing this method, the most relevant and structurally significant proteins were highlighted. These maximum values were then utilized in rank aggregation, providing a robust framework for ordering the re-clustered PCCs. This approach not only improves the reliability of the rankings but also aids in identifying the most promising candidates for further experimental validation, thereby streamlining the drug discovery process.

#### PROTAC docking into PPCs

The ultimate stage of MEGA PROTAC involves docking PROTAC into PPCs, resulting in the formation of ternary structures. MEGA PROTAC employs MEGADOCK with a reduced MEGADOCK-grid size parameter to perform a more comprehensive search for PROTAC poses. Finally, MEGA PROTAC produces thousands or even more PROTAC poses on different PPCs by increasing the selected cluster number and PCCs per cluster.

### Comparison with state-of-the-art methods

Numerous investigations have been conducted to ascertain ternary complex structures by using docking techniques and the subsequent reranking of predicted ternary structures. In one of the earliest studies, Drummond et al. benefited from several methodologies to increase the performance of PRosettaC^16^. These methodologies encompass PROTAC docking within protein–protein complexes, sampling of PROTAC lengths, and clustering of outputs to identify the most optimal ternary structures^16^. The PRosettaC algorithm employs PatchDock^47^ for sampling the protein–protein conformational space, followed by RosettaDock^28^ for doing local docking in the context of PROTAC. After the passage of about one year, Drummond et al. suggested protocols to generate ensembles of PROTAC-mediated ternary complexes based on their bound structures^4^. Based on the outcomes obtained, it was observed that the protein–protein docking approach exhibited the most favourable performance. The researchers employed sampling and filtering strategies to enhance the accuracy of protein–protein docking, namely in identifying ternary complexes^4^. Also, Bai et al. leveraged the existing length of knowledge on PROTAC to enhance their protocol by implementing geometric and energy filtering techniques^17^. To further improve the performance of the mentioned studies above, in the identification of ternary structure construction, Weng et al. constructed a protocol having four main steps^4^. The steps are (i) local docking of proteins having their ligands, (ii) filtering space according to interface residue, Open Babel Obenergy, and AutoDock Vina Score, (iii) refinement using RosettaDock, and (iv) rescoring using VoroMQA. Unfortunately, these methods have been performing poorly in PROTAC screening.

In addition to the restricted performance in PROTAC screening, a notable drawback of the aforementioned protocols is their reliance on slow molecular docking systems such as RosettaDock. For example, RosettaDock has been tested by monitoring Psuccess as a performance metric and run time^30^. Psuccess indicates the probability of success, defined as having an RMSD value of 2.0 Å or less for the lowest energy estimate. The metric known as Psuccess is commonly employed in protein docking to evaluate the precision and reliability of the docking outcomes quantitatively. To obtain a Psuccess rate of 60-80%, RosettaDOCK requires a computing time exceeding 800-1000 hours^30^. The numbers indicate that using RosettaDock in a PROTAC screening requires substantial time. Alternatively, a restricted number of running RosettaDock can limit the performance of PROTAC screening by preventing a comprehensive exploration of all potential options. As a result, poor performance is the main limitation in PROTAC screening for previous protocols, besides time-consuming molecular docking steps.

To improve the limited performance of previous protocols, Ignatov et al. used (i) ligand docking to the E3 ligase and to POI, (ii) creating “Half-Linker Clouds” and FFT-Based Conformational Search, (iii) Calculating smRMSD Values, (iv) Filtering for Ubiquitin Accessibility and (v) energy minimisation^21^. While the method offers a notable approach to predicting ternary complex structures by simultaneously sampling protein–protein and linker conformations, the inability to access their implementation restricts reproducibility and direct comparison. The study by Ignatov et al. is not included in our comparative analysis due to the lack of publicly available code, data, and detailed results from their work^21^. It is challenging to perform a comprehensive evaluation or benchmark MEGA PROTAC against their approach under consistent conditions without access to their datasets and validation metrics. As a result, the method has not been considered in the comparison analysis.

The other study has utilised AlphaFold (including AlphaFold2, AlphaFold3, and AlphaFold-multimer) to enhance the inadequate performance of prior methods. Nonetheless, the enhancement in performance was constrained^48^ when compared to BOTCP^14^. Therefore, the study has been excluded from the comparison analysis.

The BOTCP study has been an endeavour^14^. The performance enhancement was achieved by implementing a protocol consisting of five distinct steps^14^. The steps are (i) Input initialisation, (ii) Rigid pose sampling via Bayesian optimisation, (iii) Local optimisation with simulated annealing of the PROTAC stability score, (iv) Clustering, filtering using TCP-AIR energy and re-ranking and (v) Structural refinement^14^. The BOTCP protocol has surpassed a state-of-the-art protocol designed by Weng et al.^4^ in forming ternary structures^14^. Thus, BOTCP has been chosen as the cutting-edge technique since it proved that the performance of BOTCP outperformed Weng et al.’s protocol^14^. Also, The BOTCP method was the initial and last technique to execute the “unbound docking” approach, which prevents data leakage from input files^14^. Nevertheless, BOTCP has faced challenges regarding its efficacy in PROTAC screening, specifically concerning pre-refinement.

MEGA PROTAC has yet to use molecular dynamic simulations^14^ or RosettaDock^4^ for refinement purposes since such strategies necessitate a time span of multiple days for the construction of the ternary structures. MEGA PROTAC tries to optimise performance by improving input structure quality through such refinement techniques. Moreover, enhanced pre-refinement outcomes can also amplify the refinement procedures. Therefore, MEGA PROTAC was principally assessed by comparing its pre-refinement outcomes with those of a state-of-the-art BOTCP (The comparison with BOTCP (MD), after-refinement can be found in Supplementary Information).

### Preparation of test sets

Computational approaches are employed in molecular modelling to predict the binding mode and affinity between a protein receptor and a ligand molecule in the absence of pre-association, commonly referred to as “unbound” docking. This methodology involves predicting the optimal spatial configuration and composition of the ligand within the binding region of the receptor without any prior knowledge of their interaction. Therefore, unbound docking is commonly known as “real-life docking” because of its substantial impact on scientific investigations. Unbound docking is more challenging than redocking because of incompatible chemical interactions between the ligand molecule and the target since the difficulty resides in forecasting the manner in which a novel ligand may interact with a protein.

Forecasting the docking orientations for novel protein structures is naturally more complex than “bound docking.” This heightened complexity provides a more stringent assessment of the methods’ abilities. BOTCP was the first “unbound docking” approach for PROTAC screening. To prepare “unbound docking” input, BOTCP takes part in the ternary structure from different PDB files. As a result, these parts lack a perfect interface structure to construct the ternary structure. In other words, using PDB files lacking established ternary structures, BOTCP’s test cases perform unbound docking^14^. Essentially, BOTCP’s methodology guarantees that its software does not merely memorise established successful connections but can accurately anticipate new ternary structures by relying on fundamental principles. The emphasis on practicality in real-life situations makes test cases more critical and demanding. Following BOTCP’s “unbound” docking strategy^14^, MEGA PROTAC used the same PDB inputs, which inhibits bias in comparison analysis. Using PDB files lacking established ternary structures, BOTCP’s test cases perform unbound docking^14^. Thus, the same collection of 22 ternary structures (Table 2) was utilised within the framework of BOTCP^14^. The input from the PDB provided was used to execute protocols and compared with the given ternary structure in the study^14^. Table 2 summarises these ternary structure complexes.

**Table 2:** The literature documents 22 ternary 3D models for PROTAC ternary structures. BOTCP prepared them to conduct “unbound” docking by choosing docking components from various 3D PDB models that do not contain ternary structures^14^. In addition, the table displays the position of PROTAC on the ternary structure along with information about its net charge.

Complex	E3 ligase					POI					PROTAC		
PDB ID	Name	Template	Chain	N-term	C-term	Name	Template	Chain	N-term	C-term	Residue	Ionizable	Net charge
5T35-DA	VHL	4W9H-I	D	62	204	BRD4-2	5UEU-A	A	349	457	759	No	0
5T35-HE	VHL	4W9H-I	H	62	204	BRD4-2	5UEU-A	E	349	457	759	No	0
6BN7-BC	CRBN	4TZ4-C	**B**	48	426	BRD4-1	3MXF-A	C	44	168	RN3	Imine	0
6BOY-BC	CRBN	4TZ4-C	**B**	48	426	BRD4-1	3MXF-A	C	44	168	RN6	Imine	0
6HAX-BA	VHL	4W9H-I	**B**	62	204	SMARCA2	6HAZ-A	A	1378	1490	FWZ	Piperazine	1
6HAX-FE	VHL	4W9H-I	**F**	62	204	SMARCA2	6HAZ-A	E	1378	1490	FWZ	Piperazine	1
6HAY-BA	VHL	4W9H-I	**B**	62	204	SMARCA2	6HAZ-A	A	1378	1490	FX8	Piperazine	1
6HAY-FE	VHL	4W9H-I	F	62	204	SMARCA2	6HAZ-A	E	1378	1490	FX8	Piperazine	1
6HR2-BA	VHL	4W9H-I	B	62	204	SMARCA4	6ZS2-A	A	1449	1569	FWZ	Piperazine	1
6HR2-FE	VHL	4W9H-I	F	62	204	SMARCA4	6ZS2-A	E	1449	1569	FWZ	Piperazine	1
6SIS-DA	VHL	4W9H-I	D	62	204	BRD4-2	5UEU-A	A	349	457	LFE	Sec amine	1
6SIS-HE	VHL	4W9H-I	H	62	204	BRD4-2	5UEU-A	E	349	457	LFE	Sec amine	1
6W7O-CA	BIRC2	6W74-A	C	266	349	BTK	5P9J-A	A	396	656	TL7	Sec amine	1
6W7O-DB	BIRC2	6W74-A	D	266	349	BTK	5P9J-A	B	396	656	TL7	Sec amine	1
6W8I-DA	BIRC2	6W74-A	D	266	349	BTK	5P9J-A	A	396	656	TKY	Sec amine	1
6W8I-EB	BIRC2	6W74-A	E	266	349	BTK	5P9J-A	B	396	656	TKY	Sec amine	1
6W8I-FC	BIRC2	6W74-A	F	266	349	BTK	5P9J-A	C	396	656	TKY	Sec amine	1
6ZHC-AD	VHL	4W9H-I	A	62	204	BCL2L1	4QVX-A	D	2	197	QL8	Carboxylic acid	-1
7JTO-LB	VHL	4W9H-I	L	62	204	WDR5	4QL1-A	B	32	333	VKA	2 piperazines	2
7JTP-LA	VHL	4W9H-I	L	62	204	WDR5	4QL1-A	A	32	333	X6M	Piperazine	1
7KHH-CD	VHL	4W9H-I	C	62	204	BRD4-1	3MXF-A	D	44	168	WEP	No	0
7Q2J-CD	VHL	4W9H-I	C	62	204	WDR5	4QL1-A	D	32	333	8KH	Piperazine	1

### Performance evaluation

Following BOTCP^14^, the DockQ score^49^ has been used as the primary evaluation metric for our pipeline. This incorporation permits a comprehensive evaluation of the protocol’s efficiency and precision by offering a standardised metric for appraising the calibre of protein docking predictions.

- $$0< \text {DockQ} < 0.23$$—Incorrect
- $$0.23 \le \text {DockQ} < 0.49$$—Acceptable quality
- $$0.49 \le \text {DockQ} < 0.80$$—Medium quality

The DockQ score has been used to validate MEGA PROTAC’s performance and compare MEGA PROTAC with the state-of-the-art approach, BOTCP. Besides quality assessment using the DockQ score, the method’s ranking performance has also been evaluated based on its success in ranking the cluster containing the predicted complex with the highest DockQ and the rank of the cluster containing the first complex with an acceptable DockQ.

As for the other metrics used in BOTCP^14^, (i) % Near-Native and (ii) Cluster numbers. The % near-native is the ratio of proteins having $>=$ 0.23 DockQ in a cluster. (i) The % of near-native structures can indicate the practical applicability of approaches, as a higher % of near-native structures suggests a greater likelihood of selecting an appropriate structure from the cluster. (ii) The low cluster numbers demonstrate a successful filtration step by filtering out unpromising clusters. Additionally, the small number of clusters suggests that this method may be more feasible for future research, as a restricted number of clusters may be examined manually.

## Results and discussion

MEGA PROTAC has been tested on 22 existing ternary structures and compared to the state-of-the-art method, BOTCP. The comparison analysis primarily focused on three criteria: (i) the quality assessment using the DockQ score, (ii) the ranking performance assessment, and (iii) the practical usage of programs. (i) The predicted proteins’ quality assessment used the DockQ score. (ii) The ranking lists for each program have been compared. (iii) Lastly, the initial satisfactory ranking performance of BOTCP and MEGA PROTAC has been compared. Following a thorough comparison analysis with BOTCP, a case study was undertaken to identify the mechanism by which MEGA PROTAC produces its best-scoring ternary structure predictions. Finally, a case study has been finalised to demonstrate the utilisation of MEGA PROTAC and its potential outcomes.

### Comparison analysis

The primary objective of MEGA PROTAC is to enhance performance at the pre-refinement stage. Therefore, the primary analysis involves comparing the pre-refinement outcomes of MEGA PROTAC with those of BOTCP. Nevertheless, to investigate the performance of MEGA PROTAC in-depth, the findings of MEGA PROTAC were compared with the molecular dynamic simulation data obtained by BOTCP (Supplementary Information).

#### Pre-refinement performance comparison

The comparative analysis of MEGA PROTAC and BOTCP (pre-refinement) consists of three primary sections: (i) the quality assessment using the DockQ score, (ii) the ranking performance assessment, and (iii) the practical usage of programs. (i) The quality evaluation offers valuable information about the filtration system’s performance, namely its ability to retain the highest qualified protein complexes during all filtration operations. (ii) Moreover, assessing model performance by ranking is essential since it restricts the practical use of approaches when identifying the first appropriate protein structure in a low-ranked cluster. (iii) An excellent method should excel at quality and ranking concurrently, directly affecting usage in further PROTAC screening.

*The quality assessment using DockQ score* In Table 3, When evaluated using DockQ scores, the pre-refinement version of BOTCP demonstrated superior performance in three out of 22 cases (6HAY-FE, 6W8I-EB, and 7Q2J-CD), leading to an improvement in the protein’s classification, compared to MEGA PROTAC. Table 3 indicates that both techniques yielded the same categorisation results for 9 out of 22 cases. Conversely, MEGA PROTAC demonstrated superior performance in 10 out of the total test instances. MEGA PROTAC outperformed BOTCP in classification, achieving better results in 10 cases compared to BOTCP’s 3 cases, demonstrating more than three times the effectiveness. As a result, the figure illustrates that MEGA PROTAC enhanced protein quality classification performance, as indicated by the DOCKQ scores.

**Table 3:** The table displays the highest DOCKQ scores achieved by a single ternary structure output by each pipeline BOTCP (pre-refinement) and MEGA PROTAC on each of the 22 test cases.

PDB ID	BOTCP (Pre-refinement)					MEGA PROTAC					Max DockQ
	f(nat)	I-RMSD	L-RMSD	DockQ	Class	f(nat)	I-RMSD	L-RMSD	DockQ	Class 1	
5T35-DA	0.818	1.97	5.192	0.638	M	**1**	**1.361**	**4.46**	**0.778**	M	0.85
5T35-HE	0.265	2.029	8.617	0.37	A	**1**	**1.392**	**4**	**0.785**	**M**	0.88
6BN7-BC	0.286	**3.101**	7.441	0.347	A	**1**	3.543	**7.102**	**0.58**	**M**	0.8
6BOY-BC	0.568	**2.59**	10.111	0.411	A	**0.9**	2.926	**6.111**	**0.589**	**M**	0.81
6HAX-BA	0.684	**2.436**	**5.348**	**0.558**	M	**1**	2.486	11.479	0.54	M	0.85
6HAX-FE	0.444	**1.444**	**8.772**	**0.483**	A	**0.667**	6.787	17.335	0.302	A	0.85
6HAY-BA	0.786	**0.972**	**2.977**	**0.794**	M	**1**	4.216	7.585	0.556	M	0.89
6HAY-FE	**0.8**	**1.866**	**3.033**	**0.693**	**M**	0.333	4.234	12.669	0.252	A	0.87
6HR2-BA	0.45	**2.731**	14.466	0.313	A	**1**	4.317	**10.194**	**0.506**	**M**	0.81
6HR2-FE	0.625	**2.728**	14.449	0.371	A	**1**	4.393	**10.415**	**0.501**	**M**	0.84
6SIS-DA	0.7	1.701	**5.031**	0.626	M	**0.8**	**1.403**	6.97	**0.644**	M	0.86
6SIS-HE	0.458	2.365	4.76	0.502	M	**1**	**1.509**	**4.727**	**0.754**	M	0.83
6W7O-CA	0.333	3.278	6.647	0.376	A	**1**	**2.957**	**5.682**	**0.632**	**M**	0.84
6W7O-DB	0.476	**2.585**	**7.988**	0.42	A	**0.818**	3.626	11.341	**0.441**	A	0.83
6W8I-DA	0.188	2.393	**4.402**	0.419	A	**1**	**1.894**	5.359	**0.7**	**M**	0.84
6W8I-EB	0.565	**1.484**	**3.649**	**0.638**	M	**0.625**	2.281	7.857	0.489	A	0.79
6W8I-FC	**1**	5.88	28.805	0.38	A	**1**	**1.931**	**9.789**	**0.602**	**M**	0.86
6ZHC-AD	0.053	**3.42**	**5.545**	0.305	A	**1**	3.766	23.586	**0.417**	A	0.91
7JTO-LB	**0.5**	3.05	14.82	0.31	A	0.286	**2.287**	**9.741**	**0.34**	A	0.76
7JTP-LA	0.7	2.694	7.715	0.495	A	**1**	**1.856**	**6.57**	**0.674**	**M**	0.84
7KHH-CD	0.333	3.771	15.829	0.231	A	**0.8**	**1.284**	**2.974**	**0.756**	**M**	0.92
7Q2J-CD	0.385	**1.463**	**3.165**	**0.592**	**M**	**0.5**	3.694	9.617	0.36	A	0.88
**Mean**	0.519	**2.543**	**8.58**	0.467	A	**0.851**	2.916	8.889	**0.554**	**M**	0.846
**Median**	0.488	**2.511**	**7.044**	0.42	A	**1**	2.706	7.721	**0.568**	**M**	0.845

It also displays f(nat), I-RMSD, and L-RMSD values, which indicate the approaches’ prediction quality. The DockQ score has been used to determine the quality class, as shown in the Class columns. H represents high quality, M shows medium quality, and A shows acceptable quality. The final number represents the highest maximum DockQ score determined in BOTCP, indicating the maximum achievable DockQ score. They aligned unbound inputs to a native complex and computed the maximum DockQ score, achievable using rigid docking^14^.

Significant are in value [bold].

Regarding the examination of individual DockQ scores, BOTCP yields a higher DockQ score for only 5 out of 22 cases, specifically 6HAX-BA, 6HAX-FE, 6HAY-BA, 6HAY-FE, and 7Q2J-CD. The BOTCP results exhibit higher DockQ scores, ranging from 0.018 to 0.441. The most significant disparity arises from 6HAY-FE, where BOTCP yielded a DockQ score of 0.693, whereas MEGA PROTAC achieved a DockQ score of 0.252. Fortunately, MEGA PROTAC yielded a higher DockQ score for 17 of 22 proteins. The enhanced DockQ score varies between 0.018 and 0.525. The most significant enhancement, 0.525, has been reported for 7KHH-CD. As for the highest DockQ enhancement, BOTCP had the highest DockQ value of 0.231, whereas MEGA PROTAC yielded a DockQ score 0.756. The improvement in DockQ scores, 77.273% of test sets, demonstrated that MEGA PROTAC outperformed BOTCP in terms of overall results.

Regarding the assessment of overall performance utilising mean and median for DockQ score (Table 3), BOTCP yielded a mean DockQ score of 0.467 and a median score of 0.420. The values show that BOTCP demonstrated acceptable overall performance, as they are below 0.49. Fortunately, MEGA PROTAC has a mean of 0.554 and a median of 0.568. Enhancing the DockQ score is enough to elevate the total classification performance from an acceptable level to a medium one. The higher mean and median values (Table 3) for MEGA PROTAC indicate superior quality classification performance compared to BOTCP.

The other supplementary quality assessment depends on f(nat), I-RMSD, and L-RMSD values for the protein with the highest DockQ score. A higher f(nat) value indicates a higher proportion of successfully predicted contacts, which suggests a more accurate docking prediction. F(nat) is critical as it offers a valuable understanding of the structural resemblance between the anticipated and empirically determined complexes. This information is essential for comprehending the biological significance of the expected interaction. The average value of f(nat) for BOTCP is 0.512, but MEGA PROTAC yields a value of 0.851 (Table 3). The notable enhancement demonstrates a consistent pattern, with MEGA PROTAC surpassing BOTCP. As for L-RMSD, a lower I-RMSD value signifies a tight resemblance between the predicted complex and the native complex regarding the arrangement of interface residues. Lower values for L-RMSD and I-RMSD imply that the anticipated binding mechanism is more precise and probable to depict a physiologically significant interaction. BOTCP achieved a lower I-RMSD of 2.543 compared to MEGA PROTAC’s 2.916. A smaller L-RMSD value suggests a higher structural similarity between the anticipated and native ligands. This is significant as it implies that the anticipated manner in which a ligand binds is more precise, which is essential for comprehending the ligand-binding process and developing drugs. Furthermore, the MEGA PROTAC yielded a mean and median for L-RMSD of 0.3-0.6 units greater than BOTCP. The limited flexibility of MEGA PROTAC may be the primary factor hindering its ability to surpass BOTCP. As a result, MEGA PROTAC shows competitive performance with BOTCP, depending on f(nat), I-RMSD, and L-RMSD.

Overall, MEGA PROTAC demonstrated superior classification performance compared to BOTCP on 40.909% of the test sets, whereas BOTCP only exhibited higher performance on 13.636%. Also, MEGA PROTAC outperformed BOTCP by achieving a higher DockQ score in 77.273% of the test sets. Furthermore, the performance improvement was evidenced by higher mean and median scores on the DockQ scale. The greater mean and median indicate that MEGA PROTAC provided medium quality, while BOTCP provided acceptable quality. Supplementary quality metrics indicated that both MEGA PROTAC and BOTCP exhibit comparable performance. Upon careful examination of all the aforementioned outcomes, it becomes evident that MEGA PROTAC yielded superior and more competent structures than BOTCP. However, locating the more highly qualified models within the reasonable ranks is advisable to enhance the practicality of the techniques. Hence, the second comparison of approaches has been concluded based on their rankings.

*The ranking performance assessment* Previous research has employed two distinct evaluation methodologies for cluster ranking: (i) assessing the ranking performance based on the protein with the greatest DockQ score and (ii) evaluating the ranking performance based on the first acceptable protein structure ($>=$0.23 DockQ). Thus, both ranking evaluation methodologies were included in our comparative analysis. Table 4 provides a summary of cluster ranking performance with respect to the predicted complex with the best DockQ score, and Table 5 provides an overview with respect to the first acceptable ternary structure.

**Table 4:** The table displays the performance rankings for clusters containing the predicted ternary structure with the highest DockQ score.

PDB ID	BOTCP (pre-refinement)			MEGA PROTAC		
	Cluster rank	Total cluster number	% Near-native	Cluster rank	Total cluster number	% Near-native
5T35-DA	76	765	**94.1**	**7**	**59**	71.818
5T35-HE	51	763	**88.9**	**13**	**66**	37.500
6BN7-BC	89	917	6.7	**2**	**70**	**74.282**
6BOY-BC	19	720	80	**4**	**93**	**97.143**
6HAX-BA	**8**	816	**100**	**8**	**85**	82.051
6HAX-FE	14	795	**100**	**3**	**107**	32.099
6HAY-BA	**2**	914	**100**	36	**94**	**100**
6HAY-FE	**5**	946	**100**	60	**85**	14.286
6HR2-BA	73	727	**41.7**	**6**	**90**	12.121
6HR2-FE	69	679	25	**35**	**90**	**26.667**
6SIS-DA	71	339	**100**	**4**	**72**	53.333
6SIS-HE	**8**	365	**100**	17	**60**	55.844
6W7O-CA	**12**	269	9.4	62	**92**	**100**
6W7O-DB	**4**	271	29.2	58	**62**	**100**
6W8I-DA	13	362	**96.6**	**11**	**82**	66.667
6W8I-EB	**6**	364	**25**	32	**60**	16.667
6W8I-FC	35	365	**84.6**	**1**	**65**	52.381
6ZHC-AD	**7**	670	75	29	**54**	**90.909**
7JTO-LB	**15**	403	**50**	27	**75**	31.818
7JTP-LA	42	170	**88.1**	**4**	**97**	74.257
7KHH-CD	**9**	None	None	12	**65**	**65.274**
7Q2J-CD	82	322	79.1	**14**	**93**	11.429
Mean	32.273	568.667	**70.162**	**20.227**	**78**	57.570
Median	14.500	670.000	**84.600**	**12.5**	**78.5**	60.559

Mean and median values for each metric have been calculated to provide a general overall rating. The table displays the cluster ranking for MEGA PROTAC and BOTCP. Also, the total cluster number has been demonstrated. Finally, the near-native percentage demonstrates the proportion of acceptable protein in that specific cluster.

Significant are in value [bold].

Table 4 presents the total number of clusters for each set of tests. The cluster number unequivocally indicates that MEGA PROTAC yielded higher DockQ scores by examining clusters that were 7.2 times smaller on average. The fact that the cluster counts are 7.2 times less indicates our results have been in a high degree of structural similarity, which may represent successful filtration to keep more focused outputs. Conversely, the presence of clusters that are 7.2 times smaller may explain why MEGA PROTAC did not obtain high-quality results for certain tests, such as 7Q2J-CD. The reason for this could be that our grid search has limitations that prevent it from including a protein with a perfect DockQ score of 1.

MEGA PROTAC may contain structures that display a significant level of resemblance to one another. This has the potential to result in certain clusters having a significantly high percentage of individuals having a higher %Near-native, such as 6BOY-BC (97.1%), 6HAY-BA (100%), 6W7O-CA (100%) and 6W7O-DB (100%). MEGA PROTAC can exclude specific structures that do not exactly match a particular group, as decided by FCCs. These excluded structures, which could be more diverse, might exhibit reduced degrees of resemblance to native structures. Therefore, the search space of MEGA PROTAC may demonstrate a higher level of variety than that of BOTCP. In conclusion, unfortunately, BOTCP provided a higher mean and median for %Near-native for the cluster than MEGA PROTAC. Hence, the disadvantages of MEGA PROTAC shall be thoroughly explored and addressed for improvement.

Regarding rank performance for the cluster with the greatest DockQ (Table 4), MEGADOCK outperformed the other methods in 12 cases out of 22 test instances. However, in one example (6HAX-BA), the rank was the same for all proteins. MEGA PROTAC yielded a 54.545% improvement in ranking, whereas BOTCP resulted in a 40.901% enhancement in ranking performance for the cluster with the highest DockQ score. However, the statistical measures of mean and median for the ranking of the approaches (Table 4) clearly indicate that MEGA PROTAC outperforms BOTPC regarding ranking performance. MEGA PROTAC had a mean ranking of 20.227 and a median ranking of 12.5, whereas BOTPC had a mean ranking of 32.273 and a median ranking of 14.500. MEGA PROTAC demonstrated a significant improvement in the mean ranking by 37.325% and a notable improvement of 13.793% in the median ranking, as shown in Table 4.

BOTCP used TCP-AIR score to filter out clusters, and the filtered-out cluster number has been demonstrated in Table 5. The BOTCP had a mean of 249.136 and a median of 197.500 for the total cluster counts. MEGA PROTAC notably decreased average and middle values, with a mean of 78 and a median of 78.5. The results indicated that MEGA PROTAC yielded a threefold decrease in cluster numbers compared to BOTCP due to its more focused outputs. The more focused results suggest that MEGA PROTAC possesses a superior filtration approach compared to BOTCP.

**Table 5:** The table presents the performance rankings for clusters that have at least one acceptable DockQ score ($>=$0.23). Mean and median performances have been computed to better understand the overall rating. The table presents the ranking for MEGA PROTAC and BOTCP clusters. Furthermore, the overall number of clusters has been illustrated. Ultimately, the near-native percentage indicates the ratio of acceptable protein within that particular cluster.

PDB ID	BOTCP (pre-refinement)			MEGA PROTAC		
	First Acc. cluster num.	Total cluster number	%Near-native	First Acc. cluster num.	Total cluster number	%Near-native
5T35-DA	5	370	0.84	**2**	**59**	**54.198**
5T35-HE	**2**	315	31.45	7	**66**	**53.097**
6BN7-BC	30	606	3.33	**1**	**70**	**84.615**
6BOY-BC	12	423	**77.78**	**3**	**93**	73.481
6HAX-BA	**7**	300	1.04	**7**	**85**	**4.667**
6HAX-FE	6	283	0.14	**3**	**107**	**32.099**
6HAY-BA	**3**	374	**98.94**	18	**94**	44.643
6HAY-FE	**1**	377	**30.47**	19	**85**	5.085
6HR2-BA	5	302	1.11	**1**	**90**	**75**
6HR2-FE	**4**	327	**0.6**	5	**90**	0.41
6SIS-DA	**1**	161	17.76	4	**72**	**53.333**
6SIS-HE	**1**	189	2.35	7	**60**	**58.025**
6W7O-CA	**1**	102	**35.79**	4	**92**	3.03
6W7O-DB	**1**	106	**17.24**	2	**62**	0.225
6W8I-DA	**5**	191	**16.67**	**5**	**82**	3.623
6W8I-EB	**93**	196	**4**	**2**	**60**	3
6W8I-FC	**1**	188	8.7	**1**	**65**	**52.381**
6ZHC-AD	**8**	194	**5.9**	14	**54**	1.538
7JTO-LB*	4	199	**4.49**	**1**	**75**	0.149
7JTP-LA	2	49	0.43	**1**	**97**	**49.4**
7KHH-CD	3	160	0.44	**1**	**65**	**4.02**
7Q2J-CD	8	69	1.1	**2**	**93**	**1.327**
Average	9.227	249.136	16.39	**5**	**78**	**29.879**
Median	4	197.5	4.245	**3**	**78.5**	**18.592**

Significant are in value [bold].

The other important metric is %Near-native, and MEGA PROTAC showed a much greater %Near-native for clusters with at least one acceptable structure (DockQ score $>=$ 0.23). BOTCP yielded a mean of 16.390 and a median of 4.245, whereas MEGA PROTAC yielded a mean of 29.879 and a median of 18.592. The data shows that MEGA PROTAC had a mean of roughly 1.8 times greater and a median of around 4.4 times greater than BOTCP. The greater %Near-native indicates that MEGA PROTAC provides more acceptable proteins in the selected clusters. Nevertheless, in order to confirm the superior performance of MEGA PROTAC, it is necessary to prioritise these promising clusters and place them at the top of the rankings.

The other vital ranking performance is defining the first acceptable protein at the top of the rank Table 5. The mean and median of the ranks for the cluster having the first acceptable structure rank for MEGA PROTAC are 5 and 3, respectively. In contrast, the mean and median for BOTCP are 9.227 and 4.000. The 25 to 60% improvement in the mean and median indicates that MEGA PROTAC outperforms BOTCP regarding ranking performance. The better ranking and a higher %Near-native proficiency ensure that MEGA PROTAC is considerably more practical than BOTCP with the higher performance.

*The practical usage impact of programs* Theoretical ranking performance for the programs can be assessed by dividing the ranking by the total cluster. According to the approach, BOTCP mostly provided better theoretical ranking performance than MEGA PROTAC (Tables 4 and 5). Nevertheless, such a ratio has the potential to be misleading. For example, a method that can precisely identify the correct ternary structure from a million possibilities within the top thousand ranks represents the highest theoretical ranking compared to BOTCP and MEGA PROTAC. However, the discovery of suitable proteins at a thousand ranks does not have any practical implications. Thus, a better ranking is a more accurate reprranking’s performance of the ranking and impacts PROTAC screening.

**Fig. 6:**
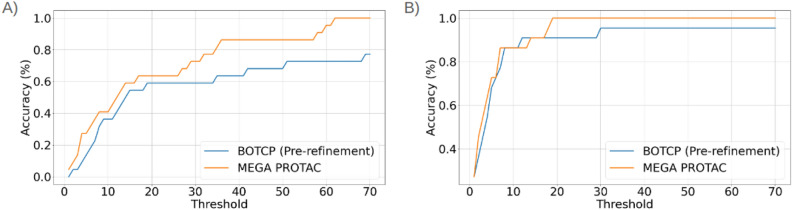
The figure illustrates the accuracy of two methods, BOTCP (pre-refinement) and MEGA PROTAC, at various thresholds. Each threshold corresponds to a ranking value, and any value lower than the threshold is considered correct. Accuracy is determined by evaluating 22 ranking results from the programs. For a given threshold, any rank that is less than or equal to the threshold is considered correct. The number of correct cases is then divided by the total number of tests, which is 22, to calculate the accuracy. “A” represents the accuracy performance for the cluster with the highest DockQ score, while “B” represents the ranking accuracy for the cluster with at least one acceptable PPC ($>=$0.23 DockQ score).

MEGA PROTAC and BOTCP must identify sufficiently better ranks to enhance practical applicability. The accuracy (as shown in Fig. 6) has been computed for each threshold to evaluate the influence of different methods on practical application. Figure 6 A illustrates the precision of cluster ranking based on the DockQ score with the highest value. MEGA PROTAC, displayed in yellow, consistently exhibited superior precision compared to BOTCP, displayed in blue. When the threshold reaches approximately 40, the difference in accuracy between the approaches becomes more evident. MEGA PROTAC has enhanced the ranking performance by over 20% for the threshold value 40. Based on a comprehensive analysis of Fig. 6 A, it can be concluded that MEGA PROTAC exhibits superior ranking performance compared to BOTCP.

Figure 6 and Table 5 demonstrate the ranking performance of clustering with at least one acceptable protein–protein complex (PPC) ($>=$0.23 DockQ score). Regrettably, the BOTCP algorithm assigned a rank of 93 to the first acceptable structure of 6W8I-EB. The 93rd cluster is highly likely to be ignored in the practical usage of BOTCP since the 93rd cluster is too enormous to be searched manually. Therefore, it could be a failure of the BOTCP in practical usage. Furthermore, BOTCP found an acceptable structure at the 30th rank for 6BN7-BC. Such unpromising ranks reduce BOTCP’s practical usage. Fortunately, MEGA PROTAC ratings are either equal to or below 20. The data indicates that MEGA PROTAC is a more feasible and robust option for use in research studies.

Figure 6B demonstrates that MEGA PROTAC, highlighted in yellow, exhibits a slight superiority over BOTCP in identifying a cluster containing at least one PPC ($>=$0.23 DockQ score) that is deemed acceptable. Nevertheless, the level of proficiency in BOTCP is noticeably lower than that of MEGA PROTAC (as shown in Table 4), making even a slightly higher rating relevant in practical applications. For example, MEGA PROTAC yielded a somewhat higher ranking for a cluster, with 29.879% of acceptable ternary structures (Table 4), whereas BOTCP resulted in a slightly lower ranking for a cluster, with 16.390% (Table 4). Therefore, MEGA PROTAC stands out as the superior option for PROTAC screening due to its elevated and more resilient overall performance.

#### Summary of comparison analysis

In summary, the major findings are:

- MEGA PROTAC demonstrated superior classification performance compared to BOTCP on 40.909% of the test sets, whereas BOTCP only exhibited higher performance on 13.636% (Table 3).
- The increase in DockQ score, with 77.273% accuracy on the test sets (Table 3), indicates that MEGA PROTAC performed better than BOTCP in terms of overall outcomes. The enhancement in DockQ elevates the mean and median quality level from acceptable to medium (Table 3).
- The mean and median ranks for the highest DockQ score were 32.273 and 14.500 for BOTCP, whereas they were 20.227 and 12.5 for MEGA PROTAC. The mean rank has improved by 37.325%, while the median rank has improved by 13.793%.
- MEGA PROTAC improved the ranks for the initial acceptable DockQ score by nearly double based on the mean and median of ranks (Table 5). Hence, MEGA PROTAC exhibits considerable promise for practical usage for PROTAC screening.
- MEGA PROTAC provided from five- to seven-fold more focused outputs based on lower clustering numbers (Tables 4 and 5). Therefore, the filtering capability of MEGA PROTAC is significantly superior to that of BOTCP.
- The performance of MEGA PROTAC and BOTCP (pre-refinement) has been evaluated based on two ranking criteria: (i) the ranking of the highest DockQ score and (ii) the ranking of the first acceptable structure. For the comparison, three metrics have been utilised: (i) ranking, (ii) total cluster number, and (iii) the percentage of Near-native in the cluster. In total, 12 assessments of the method’s rankings were conducted (2 rankings criteria * 3 metrics * 2 mean and median). MEGA PROTAC surpassed BOTCP in performance nine times, while BOTCP showed superior performance in two instances and equal performance on one occasion. In terms of overall ranking performance, MEGA PROTAC surpasses BOTCP by 75%.

In summary, the run time comparison analysis has not thoroughly compared to MEGA PROTAC because of BOTCP’s unavailability. Nevertheless, BOTCP (pre-refinement) experiences advantages from laborious procedures, such as TCP-AIR, which requires approximately 16 hours to complete for a given structure^46^. However, MEGA PROTAC can complete the entire pipeline within 4 to 8 hours, while BOTCP cannot finish calculating TCP-AIR for cluster filtration as one step of BOTCP’s pipeline. Thus, MEGA PROTAC performs notably quicker than BOTCP (pre-refinement). Furthermore, the aforementioned significant findings indicate that MEGA PROTAC exhibited superior structural quality and ranking compared to BOTCP (pre-refinement). As a result, MEGA PROTAC is considerably more convenient than BOTCP (pre-refinement).

### MEGA PROTAC performance analysis

Comprehending the reasons behind the superior quality and ranking achieved by MEGA PROTAC is a substantial advancement for future research. Hence, the performance of MEGA PROTAC is examined in three areas: (i) MEGADOCK complex analysis, (ii) assessment of the filtration of MEGA PROTAC, and (iii) assessment of rank aggregation.

#### MEGADOCK complex analysis

To examine the influence of the MEGADOCK pre-grid refinement candidate PPCs on the DockQ score, the pre-grid refinement candidate PPCs for the protein with the highest DockQ have been analyzed. Table 6 displays the variations in the DockQ score for the protein throughout our grid search, which starts from the MEGADOCK pre-grid refinement candidate PPCs to the selected protein after grid searches. Before translating MEGADOCK seeds, the mean and median values for the DockQ score were 0.308 and 0.198, respectively. Following the translation of those proteins, the mean and median values become 0.309 and 0.258, respectively. Surprisingly, no substantial improvement was observed based on these mean and median values. When the individual investigation of DockQ scores was conducted, it was found that the impact of translation on DockQ was quite restricted. Since translation and rotation are integral components in achieving a higher DockQ score, both must be accurately aligned simultaneously to attain a high score. For example, if translation is the cause of a low DockQ score, rotating each Euler angle will not alter the DockQ score. The influence of the DockQ score is minimal, thus indicating that rotating the ligand-protein is unnecessary. However, the indication about rotation can be deceptive since the actual reason why the DockQ score is low is because of translation. This also applies to the opposite scenario. Any potential translation will not enhance the DockQ score if the rotation is incorrect. So, conducting a comprehensive search for all potential translations has minimal influence on the DockQ score and does not provide evidence that the translation is redundant. A high DockQ score can only be attained if the translation and rotation are exact enough. As a result, translated structures may come closer to the native structure but with some rotational error, which may limit the DOCKQ score.

**Table 6:** The table displays the DockQ scores for the particular structure that achieved the highest DockQ score after completing the MEGA PROTAC protocol.

PDB ID	MEGADOCK	After translation	After rotation	% of improvement
5T35-DA	0.546	0.486	**0.778**	**42.491**
5T35-HE	0.555	0.507	**0.785**	**41.441**
6BN7-BC	0.420	0.360	**0.580**	**38.095**
6BOY-BC	0.487	0.353	**0.589**	**20.945**
6HAX-BA	0.304	0.450	**0.540**	**77.632**
6HAX-FE	0.080	0.078	**0.302**	**277.5**
6HAY-BA	0.192	0.260	**0.556**	**189.583**
6HAY-FE	0.095	0.131	**0.252**	**165.263**
6HR2-BA	0.204	0.199	**0.506**	**148.039**
6HR2-FE	0.191	0.193	**0.501**	**162.304**
6SIS-DA	0.575	0.662	**0.644**	**12**
6SIS-HE	0.564	0.495	**0.754**	**33.688**
6W7O-CA	**0.659**	0.606	0.632	-4.097
6W7O-DB	0.082	0.206	**0.441**	**437.805**
6W8I-DA	0.161	0.149	**0.700**	**334.783**
6W8I-EB	0.181	0.270	**0.489**	**170.166**
6W8I-FC	0.079	0.065	**0.602**	**662.025**
6ZHC-AD	0.182	0.255	**0.417**	**129.121**
7JTO-LB	0.074	0.061	**0.340**	**359.459**
7JTP-LA	0.326	0.216	**0.674**	**106.748**
7KHH-CD	0.705	0.629	**0.756**	**7.234**
7Q2J-CD	0.116	0.157	**0.360**	**210.345**
**Mean**	0.308	0.309	**0.554**	**79.965**
**Median**	0.198	0.258	**0.568**	**186.869**

The DockQ score has been calculated for the particular structure of the MEGADOCK pre-grid refinement candidate PPCs and the translated temporary structure. Ultimately, the percentage of improvement has been revealed to illustrate the progress made using our grid search method based on different starting points.

Significant are in value [bold].

As anticipated, optimising two components (translation and rotation) simultaneously led to a significant increase in the DockQ score from 7% to 662%, with the exception of one structure. The grid technique only exhibits a decrease in DockQ score for one specific protein complex, 6W7O-CA. MEGA PROTAC experienced a decrease of only 5% in its DockQ score for the 6W7O-CA protein. Conversely, the mean is augmented by 79.965%, while the median is augmented by 186.869%. Also, over 50% of the test cases, specifically 12, were found to be incorrect MEGADOCK predictions based on the DockQ score (Table 6). Fortunately, MEGA PROTAC successfully rescued those 12 out of 22 test cases, so these 12 DockQ scores have been significantly improved. In addition to enhancing inaccurate MEGADOCK predictions, MEGA PROTAC preserved other favourable structures for the remaining test cases. MEGADOCK identified a structure for 7KHH-CD with a DockQ score of 0.705. MEGA PROTAC retained this favourable structure throughout the grid search. MEGA PROTAC yielded a DockQ value of 0.756 for the 7KHH-CD protein, which was higher than the MEGADOCK prediction. The fact that MEGA PROTAC can rescue unpromising proteins and maintain a promising structure during grid search demonstrates the effectiveness of its filtration and ranking approach. MEGA PROTAC’s effective filtration and ranking method greatly enhances PROTAC screening performance.

Table 7 displays the chosen translation and rotational parameters utilised to enhance the DockQ score. The initial search areas, referred to as pre-grid refinement candidate PPCs, were generated using MEGADOCK; however, in our grid study, these original MEGADOCK pre-grid refinement candidate PPCs were not directly employed. Instead, all pre-grid refinement candidate PPCs underwent modifications through translation and rotation, as detailed in Table 7. The average translation value of 1.909 Å and the median value of 1.5 Å indicate that the pre-grid refinement candidate PPC requires less translation along the Y axis than the X and Z axes. The mean and median for translation is around 25% below our maximum threshold of 4.5 Å. The figures illustrate that the translation restriction is sufficiently extensive to encompass highly potential candidates. Furthermore, the rotation values were found to be roughly 25% lower than our upper limit (15 degrees), with both the mean and median displaying this trend. This suggests that our decision to select a 15-degree rotation, based on information discovered in the literature, was reasonably accurate.

**Table 7:** The table displays the magnitude of translation and rotation for the 22 proteins with the highest DockQ score.

Protein	Translation (A)			Rotation (degree)		
	x	y	z	x	y	z
5T35-DA	− 3	3	− 4.5	− 15	− 10	− 5
5T35-HE	− 3	3	− 4.5	− 15	− 10	− 5
6BN7-BC	− 4.5	0	− 3	− 15	5	15
6BOY-BC	− 3	0	− 4.5	− 10	− 10	15
6HAX-BA	− 1.5	− 3	1.5	− 5	− 15	0
6HAX-FE	− 3	1.5	0	− 5	− 5	15
6HAY-BA	− 4.5	1.5	4.5	15	15	− 10
6HAY-FE	− 4.5	0	− 4.5	− 5	− 10	10
6HR2-BA	− 4.5	1.5	0	− 15	− 10	− 10
6HR2-FE	− 4.5	1.5	0	− 15	− 10	− 10
6SIS-DA	− 1.5	− 1.5	1.5	− 5	− 5	0
6SIS-HE	− 3	3	− 4.5	− 15	− 10	− 5
6W7O-CA	1.5	− 1.5	4.5	5	5	0
6W7O-DB	4.5	− 4.5	− 3	15	− 15	15
6W8I-DA	3	1.5	1.5	− 15	− 15	15
6W8I-EB	3	1.5	0	− 15	− 5	5
6W8I-FC	0	− 1.5	1.5	− 15	− 15	− 15
6ZHC-AD	0	− 1.5	− 1.5	− 10	− 15	− 15
7JTO-LB	− 1.5	− 1.5	3	− 10	15	− 15
7JTP-LA	− 4.5	3	4.5	− 5	− 5	− 15
7KHH-CD	1.5	− 3	0	10	− 5	15
7Q2J-CD	− 3	3	4.5	− 15	0	0
Mean	2.864	1.909	2.591	11.364	9.545	9.545
Median	3	1.5	3	15	10	10

Translation is displayed in Armstrong units, whereas rotation is shown in degrees. Furthermore, the mean and median are computed using absolute values.

After reaching satisfactory results for the grid search on translation and rotation top limits, the final choice is to employ a more extensive grid size to enhance the DockQ values for MEGA PROTAC. For instance, decreasing the angle to less than 5 degrees and reducing the grid size to less than 1.5 Å can enhance DockQ results. An alternative approach to enhance the DockQ score for MEGA PROTAC is to do iterative translational and rotational grid searches, as each iteration has the potential to boost the DockQ score by reducing angle and grid size. Nevertheless, this situation poses a quandary where one must choose between achieving high performance and maximising efficiency in terms of time.

#### Assessment of the DockQ score improvement across grid search of MEGA PROTAC

**Fig. 7:**
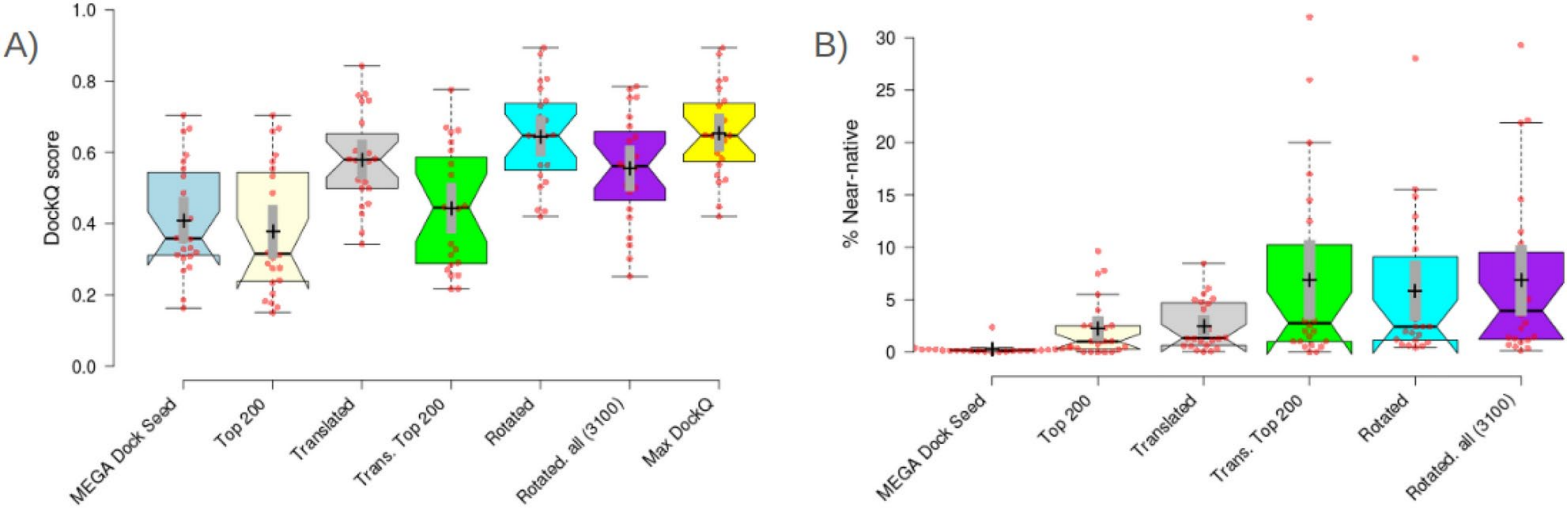
The box graphs illustrate how overall performance is affected by grid search and filtration processes. A represents that different colours for various categories represent the maximum DockQ scores: MEGADOCK pre-grid refinement candidate PPCs (light blue), selected pre-grid refinement candidate PPCs (light yellow), translated proteins (grey), selected translated proteins (green), rotated proteins (cyan), final proteins (purple), and the maximum DockQ score attained in grid search (yellow). B represents the percentages of near-native proteins with a DockQ score greater than or equal to 0.23 for each technique, in the same order. The mean is denoted by the symbol “+” and accompanied by a grey rectangle region indicating a 95% confidence interval. Ultimately, the notches illustrate the median values in box plots.

Grid search approaches should preserve promising structures during filtration and provide them as the ultimate output while managing and rectifying erroneous occurrences. To assess the performance of our grid search and filtration, two metrics were tracked across all processes of MEGA PROTAC: (i) the maximum DockQ score and (ii) the percentage of proteins near-native. Therefore, the box plots have been represented in Fig. 7.

The mean DockQ max score for MEGADOCK pre-grid refinement candidate PPCs was 0.413, which is considered acceptable quality. The mean of DockQ score for the MEGADOCK pre-grid refinement candidate PPC demonstrates that the selection of MEGADOCK was promising to successfully create initial search space as a pre-grid refinement candidate PPC. However, the mean of %Near native for the MEGADOCK pre-grid refinement candidate PPC (5000) is under 0.3% (Fig. 7B). Filtering using our sequential filtration approach and then ranking PPCs using our rank aggregation to select the most promising 200 resulted in a decrease in the mean of the DockQ maximum drop from 0.413 to 0.380 (Fig. 7A). However, selecting 200 MEGADOCK predicted structures led to a tenfold increase in the mean of %Near native results overall. Choosing a mere 4% of pre-grid refinement MEGADOCK candidate PPCs (200 PPCs) results in a lower mean of DockQ scores, deemed acceptable for the next grid search steps. MEGA PROTAC filtration and rank aggregation have shown promising results for future investigations into PROTAC ternary structure creation methods. It has demonstrated a significant 10-fold improvement in the acceptable percentage with an acceptable loss on the mean DockQ score.

When the translation grid search employed the top 200 promising MEGADOCK pre-grid refinement candidate PPCs, there was a significant increase in the mean (+) and median (notches) values of the maximum DockQ score, from 0.380 and 0.312 to 0.581 and 0.579 (Fig. 7A), respectively. Nonetheless, the mean (+) proportion of near-native complexes in the top 200 pre-grid refinement candidate PPCs (Fig. 7B) and all translated (Fig. 7B) is nearly identical, at 2.275%. Despite the significant improvement in maximum DockQ scores achieved with our transitional grid search, the mean DockQ score decreased from 0.581 to 0.443 when the top 200 translated complexes were selected after filtration. The dip signifies three potential outcomes: (i) The selected proteins (200) are insufficient to encompass all potential candidates. (ii) Our rank aggregation success is insufficient to maintain the top positions of these prospective candidates. (iii) It is most likely that the reason is a combination of these factors. As a result, conserving computer power by choosing less than 0.3% of translated PPCs that result in tolerable performance loss is worthwhile.

To do a rotational grid search, 200 translated proteins were chosen, which were used to generate rotated proteins. The maximum DockQ score obtained from the grid search and MEGADOCK is depicted in the yellow box plot in Fig. 7. It closely resembles the pre-grid refinement candidate PPCs coming from MEGADOCK outputs, which is indicated by the light blue colour. The resemblance illustrates that our grid search systematically enhances DockQ scores. Furthermore, the average value of the maximum DockQ score has risen from 0.443 to 0.646. The mean value of DockQ of 0.646 provides strong evidence that ternary structures can be built without the need for time-consuming and computationally intensive molecular dynamic simulations or RosettaDock. Nevertheless, by using our filtration rules to optimise efficiency and computational resources, the DockQ score experienced a decrease from 0.646 coloured in cyan to 0.554 coloured in purple (Fig. 7A). The figures illustrate three potential scenarios: either the filtering capability is constrained, the ranking performance is limited, or both. Conversely, our filtering has led to a minor improvement in the percentage of near-natives ($>=$ 0.23 DockQ score), as seen in Fig. 7B. As a result, the current version of MEGA PROTAC has outperformed the currently available advanced approach, BOTCP, despite its limitations.

Overall, the grid resulted in a mean improvement in DockQ from 0.413 of MEGADOCK pre-grid refinement candidate PPCs (light blue in Fig. 7A) to 0.646 (cyan in Fig. 7A) and a median increase in DockQ max hit from 0.357 (light blue on Fig. 7A) to 0.648 (cyan on Fig. 7A). Furthermore, the average near-native percentages have had a 60-fold enhancement following translational and rotational optimisation. In contrast, the median has undergone a 20-fold improvement by improving each DockQ score for MEGADOCK pre-grid refinement candidate PPC (Fig. 7B). The findings illustrate that MEGA PROTAC significantly enhances the overall performance of MEGADOCK pre-grid refinement candidate PPCs.

#### Assessment of ranking performance of individual component and rank aggregation

MEGA PROTAC employs VoroMQA and SASA in the process of rank aggregation since SASA is anticipated to be one of the most efficacious features for PROTAC. A higher SASA indicates that the protein structure has a greater surface area for binding, making it more favourable for a large PROTAC molecule to bind. However, VoroMQA has already been utilised in PROTAC design studies, and its performance has been demonstrated. Consequently, both were employed in our rank aggregation process following filtration. The section will focus on the assessment of rank aggregation in MEGA PROTAC.

**Fig. 8:**
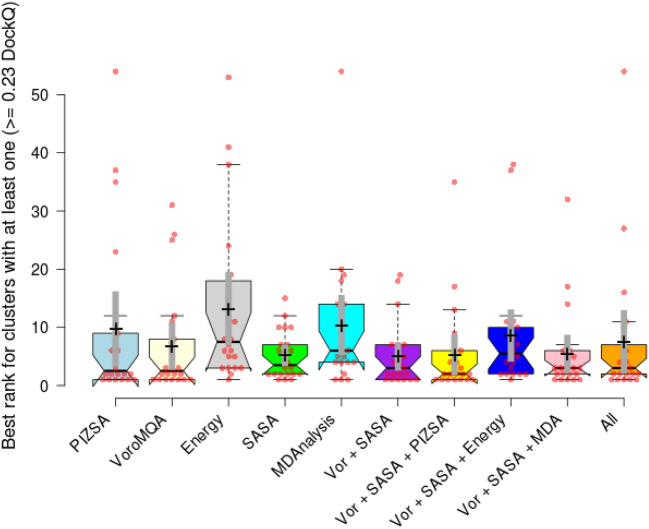
The box plots display the distribution of rankings for individual ranks and potential rank aggregations on the final outputs after the grid search of MEGA PROTAC. The X-axis displays the individual rankings and the rank aggregate name, while the Y-axis represents the rank of that cluster. MEGA PROTAC utilises five protein-based filtrations, namely PIZSA, VoroMQA, Energy, SASA, and MDAnalysis. Three-component rank aggregation approaches were demonstrated in three box plots: Vor + SASA + PIZSA coloured in yellow, Vor + SASA + Energy coloured in blue, and Vor + SASA and MDA coloured in light orange. The orange represents a rank aggregation containing “All” filtration techniques, including PIZSA, VoroMQA, Obabel (obenergy), SASA, and MDAnalysis. The optimal values have been utilised to arrange clusters, and the ranking of clusters with at least one protein structure that meets the acceptable threshold ($>=$0.23) is depicted in box plots. The mean is denoted by the symbol “+” and accompanied by a grey rectangle region indicating a 95% confidence interval. Ultimately, the notches illustrate the median values in box plots.

Figure 8 illustrates ranks of clusters that have at least one acceptable structure with a DockQ score greater than or equal to 0.23. Using energy-based filtration (Obabel) may result in losing some data based on the worst ranking performance. The poor ranking performance suggests that promising structures are located at the bottom of the ranks. Therefore, strict filtering based on energy score may result in losing these potential structures towards the end of the rankings. Fortunately, despite being used for the first time to filter ternary candidates, PIZSA, SASA, and MDA demonstrated remarkable ranking performance in identifying the first acceptable protein in a cluster (Fig. 8). SASA, in particular, demonstrated the second highest level of performance, or the highest level among individual ranking performances (Fig. 8). Thus, our overall concept of identifying the most qualified structure with the greatest distance between protein hypotheses was validated by the performance of SASA and VoroMQA, as shown in Fig. 8.

Regarding rank aggregation, the collective ranking outcomes typically outperform individual performance. More specifically, there were five combinations observed: (i) VoroMQA + SASA, (ii) VoroMQA + SASA and PIZSA, (iii) VoroMQA + SASA and MDA, (iv) VoroMQA + SASA and Energy and (v) all components used in rank aggregation (Fig. 8). First of all, the mean (+) and median (notches) show that the rank aggregation approach performs better than individual components. Also, lower variance for rank aggregation indicates that rank aggregation options are more robust than individual ranking. Once Energy became a component of rank aggregation, the mean (+) and median (notches) increased in dark blue and orange. The worse mean (+) and median (notches) demonstrate that energy-based ranking is not the best option in rank aggregation. On the other hand, three rank aggregations, (i) VoroMQA + SASA, (ii) VoroMQA + SASA and PIZSA, and (iii) VoroMQA + SASA and MDA, are promising to be used in MEGA PROTAC. The lowest mean for these three ranks can be shown for the first rank aggregation (VoroMQA + SASA). Also, 95% confidence intervals of means demonstrated with grey rectangular around “+” shows VoroMQA + SASA is slightly better than others to rank acceptable DockQ score.

**Fig. 9:**
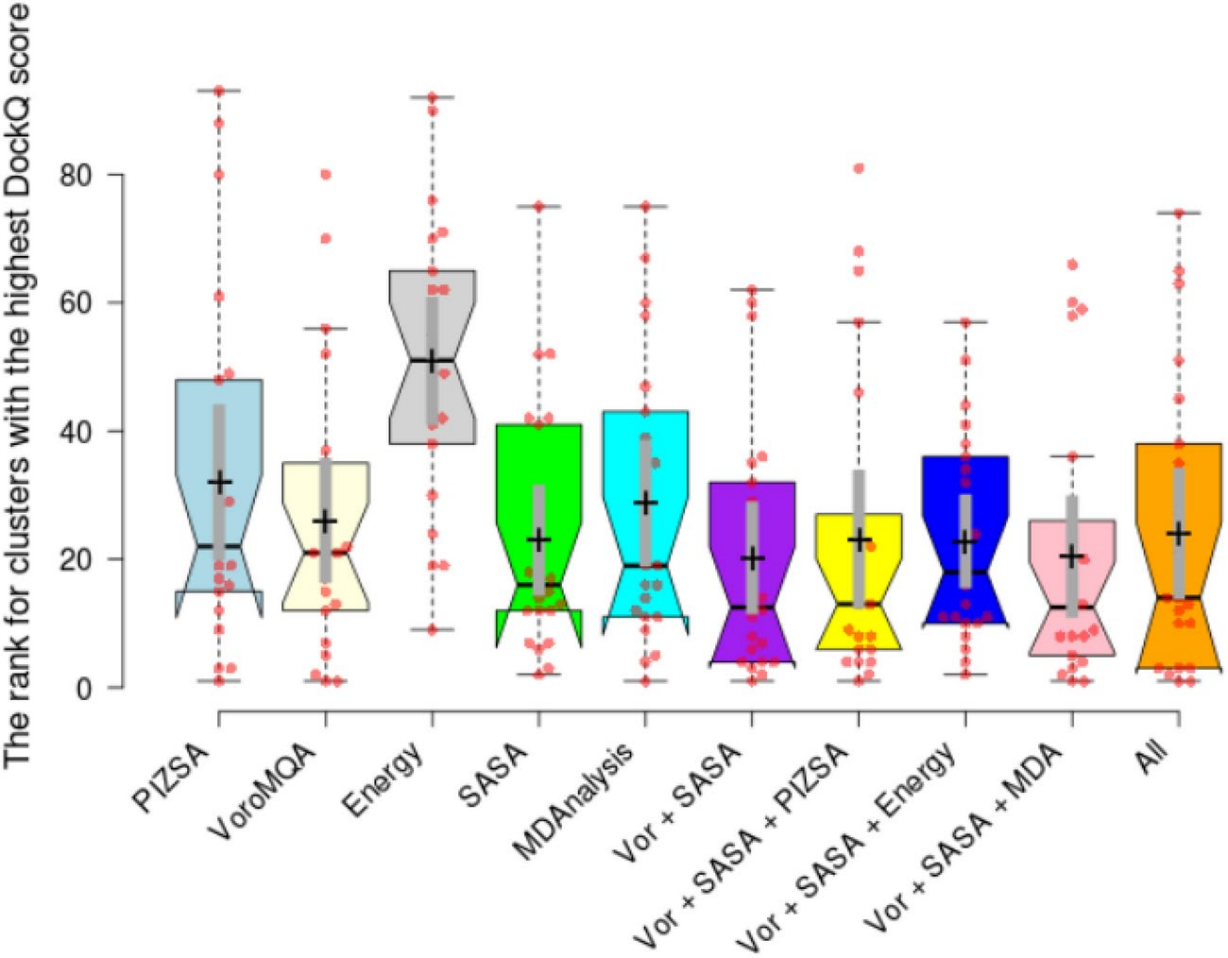
The box plots illustrate the distribution of rankings for each individual rank and the potential rank aggregations for clusters with the greatest DockQ score. The X-axis exhibits the individual rankings and the aggregate name of the rank, while the Y-axis represents the rank of that cluster. MEGA PROTAC utilises five protein-based filtrations, namely PIZSA, VoroMQA, Obabel (obenergy), SASA, and MDAnalysis. Three-component rank aggregation approaches were demonstrated in three box plots: Vor + SASA + PIZSA coloured in yellow, Vor + SASA + Energy coloured in blue, and Vor + SASA and MDA coloured in light orange. The orange represents a rank aggregation containing “All” filtration, including PIZSA, VoroMQA, Obabel (obenergy), SASA, and MDAnalysis. The mean is denoted by the symbol “+” and accompanied by a grey rectangle region indicating a 95% confidence interval. Ultimately, the notches illustrate the median values in box plots.

Figure 9 illustrates the ranking performance of MEGA PROTAC in identifying the protein with the greatest DockQ score. The individual ranking performance trends from best to worst remain consistent with earlier observations: (i) SASA or VoroMQA, (ii) MDA, (iii) PIZSA, and (iv) Energy (Obenergy). Regrettably, Obenergy assigned worse ranks to several ternary structures, impeding our proposed extra filtration. Consequently, the box plots demonstrate that using SASA and VoroMQA in the rank aggregation process effectively enhanced the ranking performance in PROTAC screening.

Regarding rank aggregation’s performance in identifying the greatest DockQ score, it is noteworthy that rank aggregation performance is generally superior or comparable to individual ranking performance (Fig. 9). As for the assessment of rank aggeration possibilities, there were five possible rank aggregation combinations observed: (i) VoroMQA + SASA, (ii) VoroMQA + SASA and PIZSA, (iii) VoroMQA + SASA and MDA, (iv) VoroMQA + SASA and Energy and (v) all components used in rank aggregation. Here, the first three possible rank aggregations were promising to rank clusters with the highest DockQ score compared to the last two possibilities. Although the first three possibilities are similar, slightly lower mean (+) and 95% confidence intervals of means (grey rectangular) demonstrate that VoroMQA + SASA rank aggregation may provide the best or competitive performance against other rank aggregation designs.

### Case study: visual inspection of ternary structure prediction performance

**Fig. 10:**
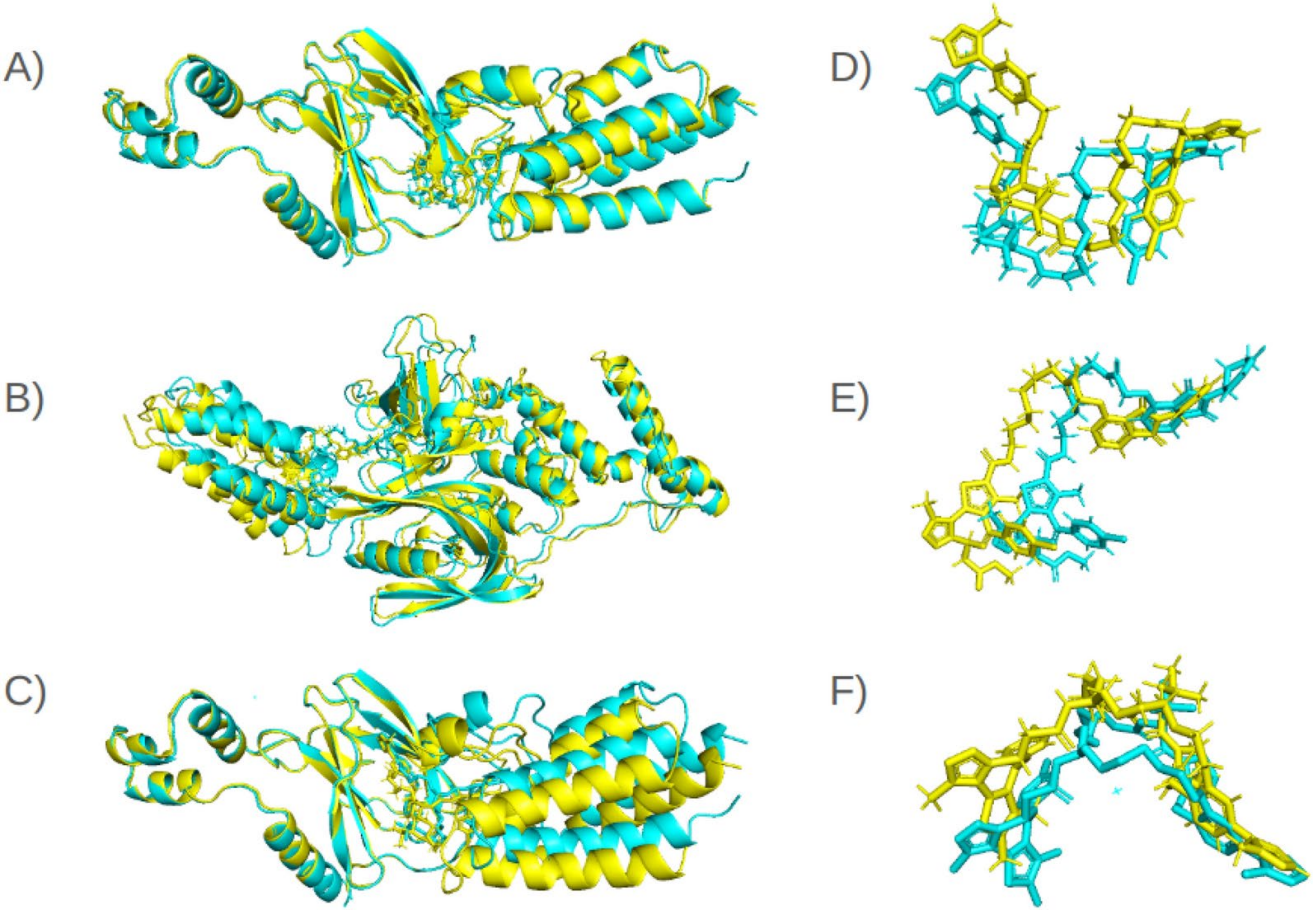
The figure illustrates three ternary structures and the corresponding poses of PROTAC for each structure. The colour yellow is used to symbolise the predicted models, whereas cyan is used to indicate the ground truth structure. A ternary structure is depicted for 5T35-DA, whereas D illustrates the visualisation of the protein’s PROTAC structure. The ternary structure and PROTAC pose are depicted in panels B and E, respectively, for the 6BN7-BC complex. The last case example showcased the ternary model and PROTAC conformation of 6SIS-DA, which were visualised in C and F, respectively.

MEGA PROTAC performs blind docking the PROTAC into protein complexes to save computer power and time using MEGADOCK instead of the other molecular docking programs evaluated in Table 1. To demonstrate the practical application and effectiveness of MEGA PROTAC, MEGADOCK was employed for randomly selected three test cases: 5T35-DA, 6BN7-BC, and 6SIS-DA (Fig. 10).

Figure 10A displays the predicted structure in yellow and the ground-truth structure in yellow for the 5T35-DA sample. The strong similarity in overall structure between the yellow and cyan structures implies that MEGA PROTAC correctly identified the ternary structure for 5T35-DA. Regarding the second ternary structure depicted in Fig. 10B, 6BN7-BC, it is worth noting that while there are mismatched beta sheets on the right and left edges of the structure, the interface between the proteins, where the PROTAC is expected to bind, has been accurately predicted. The final ternary structure for 6SIS-DA, as shown in Fig. 10C, exhibited acceptable performance. However, there were minor discrepancies in the beta sheets on the right edge of the ternary structure. MEGA PROTAC accurately predicted the majority of the remaining structure.

Figure 10 demonstrate the success of identifying PROTAC poses, highlighting why previous approaches primarily targeted highly promising protein complexes. Once the protein complexes are sufficiently good, even rapid blind docking without prior knowledge can yield high-quality PROTAC poses. Thus, similar to prior investigations, our analysis and study primarily concentrated on effectively discerning protein complexes.

### Challenges and future opportunities for MEGA PROTAC

A significant problem in the computational modelling of PROTACs is the scarcity of empirically confirmed ternary structures in the PDB. This constrains the dimensions of test sets, which may subsequently diminish the robustness of benchmarking investigations. In this analysis, we incorporated all recognised validated PROTAC structures consistent with the BOTCP study, utilising the complete dataset to guarantee a thorough and impartial evaluation. This method reduces selection bias, yet the scarcity of structural data continues to hinder the assessment of MEGA PROTAC’s broader applicability.

As more experimentally confirmed PROTAC ternary complexes become available, future research may use sophisticated multimodel feature selection methods to improve PROTAC screening^50^. The amalgamation of these methodologies, along with enhanced structural data, presents a promising avenue for augmenting MEGA PROTAC’s prediction precision and resilience, thereby enhancing its capacity to inform the advancement of next-generation PROTACs.

A significant limitation of MEGA PROTAC is the difficulty of employing AI-based technologies such as AlphaFold2 and RosettaFold to predict ternary PROTAC complexes precisely^48,51,52^. Although these tools have achieved significant progress in protein structure prediction and protein–protein interaction modelling, they remain unoptimised for the distinct complexities of PROTAC ternary structures, which require the accurate orientation of multiple proteins and ligands to predict binding stability and efficacy^48^. While theoretically possible, using these AI models without a verified framework poses a danger of producing false or deceptive structural predictions. Addressing this deficiency necessitates the creation of specialised AI models or validation methodologies specifically designed for ternary complex generation in PROTACs. In the future, incorporating AI-driven methodologies for predicting the ternary structure of PROTAC could significantly improve MEGA PROTAC’s functionalities, facilitating more thorough screening and evaluation of prospective PROTAC candidates. Until such breakthroughs are achieved, our emphasis on experimentally confirmed structures offers a more dependable foundation for optimising and ranking PROTAC setups.

MEGA PROTAC and prior methodologies utilise specified binding conformations for both the E3-anchor and POI-warhead poses, which is a significant constraint. By omitting a pre-PROTAC screening stage, using both the E3-anchor and POI-warhead poses can reduce efficacy when faced with more varied or new binding interactions. Fortunately, the binding configuration of the anchor to E3 ligases is very stable, with merely 117 identified anchor-E3 combinations, the majority of which are available in the Protein Data Bank (PDB)^53^ and PROTAC-DB^54,55^. However, a specific challenge is the unpredictability of the warhead’s binding orientation with the protein of interest (POI). The variety in warhead-point-of-interest interactions requires examining a somewhat broader conformational space, which may influence the precision and reliability of MEGA PROTAC, BOTCP, and analogous methodologies. MEGA PROTAC possesses a significant advantage in processing speed, functioning up to 60 times quicker than BOTCP (based on the run time of method components^22,23,35,36^), enabling it to adeptly manage the enlarged search space and filter protein poses with enhanced efficacy.

Future enhancements to MEGA PROTAC may augment its predicting skills for warhead-POI binding conformations, potentially incorporating sophisticated AI-driven structural tools. MEGA PROTAC’s dependence on predetermined binding postures for both the anchor and warhead facilitates consistent and impartial comparative evaluations; nevertheless, advancing computational models may allow it to accommodate a broader spectrum of PROTAC ternary structures with enhanced precision. By integrating advanced posture prediction algorithms, MEGA PROTAC could get enhanced precision, thus expanding its usefulness and efficacy in intricate PROTAC screening tasks.

In the work of Ignatov et al.^21^, simultaneous sampling of protein–protein and PROTAC interactions is implemented, enabling a more integrated approach to model ternary complex formation. This simultaneous search method is particularly advantageous regarding efficiency and accuracy, as it captures the dynamic nature of PROTAC assembly in a single step. Currently, MEGA PROTAC operates using a sequential, two-step approach that models protein–protein interactions before incorporating the PROTAC. This limitation may reduce predictive accuracy in identifying the most favourable ternary complex configurations. However, we anticipate that future versions of MEGA PROTAC will incorporate an enhanced scoring function to support simultaneous searching. This advancement would allow MEGA PROTAC to more effectively mimic the natural assembly process of ternary complexes, improving both the speed and accuracy of PROTAC screening.

A notable limitation of MEGA PROTAC is the potential loss of promising structures during the initial grid search due to rigid distance-based filtering, as shown in Fig. 7. This filtering method, based on a distance threshold between the two moieties of the PROTAC, significantly enhances computational efficiency, allowing MEGA PROTAC to evaluate thousands of candidates in seconds. In contrast, protein structure-based filtration would require several hours to process the same number of candidates, making distance-based selection a practical alternative. Our chosen range-3 Å as a lower threshold and 20 Å as an upper limit-covers nearly all structures as observed in the PRosettaC study. Although the 20 Å upper limit might exclude some optimal candidates, MEGA PROTAC is designed to prioritize finding the first acceptable structure, ensuring that a suitable complex is consistently identified for each test case. This approach progressively retains structures with higher mean and median DockQ scores, enhancing the quality of selected complexes.

For future iterations of MEGA PROTAC, we aim to address this limitation by incorporating an additional angle-based measure, specifically the angles between the mass centres of moieties and each protein mass centre. We believe that this enhancement will improve the precision of the ligand-based filtration, capturing a broader range of high-quality conformations and further boosting MEGA PROTAC’s predictive performance.

MEGA PROTAC currently employs a rigid docking approach to conserve computational resources and manage costs. However, as a rigid docking tool, it does not fully capture the dynamic nature of PROTAC structures, particularly the flexibility and conformational changes that linkers may undergo. Addressing this limitation in future versions of MEGA PROTAC could involve refining its algorithms and scoring functions to better account for linker dynamics, steric interference, and other structural factors critical for accurately modeling ternary complexes. By incorporating these aspects, MEGA PROTAC would be better equipped to predict binding efficacy in complex environments, where linker flexibility and steric hindrance significantly impact the stability and effectiveness of PROTAC interactions. Another promising direction would be to expand collaborations with experimental researchers to increase the availability of validated 3D ternary structures, providing a stronger foundation for algorithm training and validation. By integrating more advanced computational models with enriched structural datasets, MEGA PROTAC could enhance its predictive accuracy and reliability for novel PROTACs, ultimately advancing the discovery process in this rapidly evolving field.

## Conclusion

The generation of protein–protein complexes in constructing ternary structures frequently necessitates the application of protein–protein docking programs. Therefore, Table 1 presents a comprehensive overview of various docking programs that will be analysed to determine and select the construction method that exhibits higher performance.

MEGA PROTAC has been designed as a rigid docking approach using sequential filtration integrated with rank aggregation. Although MEGA PROTAC has not used any structural refinement using molecular dynamic simulations or Rosetta, MEGA PROTAC has been compared with pre-refinement results of the state-of-the-art method, BOTCP. The results demonstrate that MEGA PROTAC provided a better DockQ score in 77.273% out of 22 test cases. MEGA PROTAC effectively doubled the rank performance for the initial acceptable DockQ score. It demonstrates superior overall ranking performance, surpassing BOTCP (pre-refinement) by a significant margin of 75%.

## Supplementary Information

Supplementary Information.

## Data Availability

The input data and source codes for our research on MEGA PROTAC can be accessed on GitHub at the MEGA PROTAC repository (https://github.com/yauz3/MEGA-PROTAC). This repository includes all necessary files and scripts to replicate and further explore our study.

## Abbreviations

ML: Machine learning
PROTACs: Proteolysis-targeting chimaeras
CoBDock: Consensus blind dock
PPCs: Protein–protein complexes
BOTCP: Bayesian optimisation for ternary complex prediction
SBD: The substrate binding domain
Ub: Ubiquitin
POI: Protein of interest
TC: Ternary complex
FFT: Fast Fourier transform
SASA: Solvent accessible surface area
UFF: Universal Force field
Gaff: General AMBER force field
PPIs: Protein–protein interactions
PIZSA: Protein interaction Z-score assessment
CAPRI: Critical assessment of Protein Structure Prediction
RMSD: Root mean square deviation
MD: Molecular dynamic
RRT: Relative rotation and translation

## References

[1] Sakamoto, K. M. Chimeric molecules to target proteins for ubiquitination and degradation. *Methods Enzymol.* **399**, 833–847 (2005). 16338398 10.1016/S0076-6879(05)99054-X

[2] Schapira, M., Calabrese, M. F., Bullock, A. N. & Crews, C. M. Targeted protein degradation: Expanding the toolbox. *Nat. Rev Drug Discov* **18**(12), 949–963 (2019). 31666732 10.1038/s41573-019-0047-y

[3] Bai, N. et al. Modeling the CRL4A ligase complex to predict target protein ubiquitination induced by cereblon-recruiting PROTACs. *J Biol Chem* **298**(4), 101653 (2022). 35101445 10.1016/j.jbc.2022.101653 PMC9019245

[4] Weng, G., Li, D., Kang, Y. & Hou, T. Integrative modeling of PROTAC-mediated ternary complexes. *J. Med. Chem.* **64**(21), 16271–16281 (2021). 34709816 10.1021/acs.jmedchem.1c01576

[5] Troup, R. I., Fallan, C. & Baud, M. G. J. Current strategies for the design of PROTAC linkers: A critical review. *Explor. Target. Anti-tumor Ther.* **1**(5), 273 (2020). 10.37349/etat.2020.00018 PMC9400730 36046485

[6] Schneekloth, J. S. Jr. et al. Chemical genetic control of protein levels: Selective in vivo targeted degradation. *J. Am. Chem. Soc.* **126**(12), 3748–3754 (2004). 15038727 10.1021/ja039025z

[7] Burslem, G. M. & Crews, C. M. Small-molecule modulation of protein homeostasis. *Chem. Rev.* **117**(17), 11269–11301 (2017). 28777566 10.1021/acs.chemrev.7b00077

[8] Adams, J. The proteasome: Structure, function, and role in the cell. *Cancer Treat. Rev.* **29**, 3–9 (2003). 12738238 10.1016/s0305-7372(03)00081-1

[9] Metzger, M. B., Hristova, V. A. & Weissman, A. M. Hect and ring finger families of e3 ubiquitin ligases at a glance. *J. Cell Sci.* **125**(3), 531–537 (2012). 22389392 10.1242/jcs.091777 PMC3381717

[10] Wang, C. et al. The state of the art of PROTAC technologies for drug discovery. *Eur. J. Med. Chem.* **235**, 114290 (2022). 35307618 10.1016/j.ejmech.2022.114290

[11] Qi, S.-M. et al. PROTAC: An effective targeted protein degradation strategy for cancer therapy. *Front. Pharmacol.* **12**, 692574 (2021). 34025443 10.3389/fphar.2021.692574 PMC8138175

[12] Mullard, A. Targeted protein degraders crowd into the clinic. *Nat. Rev. Drug Discov.* **20**(4), 247–250 (2021). 33737725 10.1038/d41573-021-00052-4

[13] Gao, H., Sun, X. & Rao, Yu. PROTAC technology: Opportunities and challenges. *ACS Med. Chem. Lett.* **11**(3), 237–240 (2020). 32184950 10.1021/acsmedchemlett.9b00597 PMC7073876

[14] Rao, A. et al. Bayesian optimization for ternary complex prediction (BOTCP). *Artif. Intell. Life Sci.* **3**, 100072 (2023).

[15] Bondeson, D. P. et al. Catalytic in vivo protein knockdown by small-molecule PROTACs. *Nat. Chem. Biol.* **11**(8), 611–617 (2015). 26075522 10.1038/nchembio.1858 PMC4629852

[16] Zaidman, D., Prilusky, J. & London, N. Prosettac: Rosetta based modeling of PROTAC mediated ternary complexes. *J. Chem. Inf. Model.* **60**(10), 4894–4903 (2020). 32976709 10.1021/acs.jcim.0c00589 PMC7592117

[17] Bai, N. et al. Rationalizing PROTAC-mediated ternary complex formation using Rosetta. *J. Chem. Inf. Model.* **61**(3), 1368–1382 (2021). 33625214 10.1021/acs.jcim.0c01451 PMC8866032

[18] Lai, A. C. & Crews, C. M. Induced protein degradation: An emerging drug discovery paradigm. *Nat. Rev. Drug discov.* **16**(2), 101–114 (2017). 27885283 10.1038/nrd.2016.211 PMC5684876

[19] Bai, L. et al. A potent and selective small-molecule degrader of stat3 achieves complete tumor regression in vivo. *Cancer Cell* **36**(5), 498–511 (2019). 31715132 10.1016/j.ccell.2019.10.002 PMC6880868

[20] Liao, J., Nie, X., Unarta, I. C., Ericksen, S. S. & Tang, W. In silico modeling and scoring of PROTAC-mediated ternary complex poses. *J. Med. Chem.* **65**(8), 6116–6132 (2022). 35412837 10.1021/acs.jmedchem.1c02155 PMC13242788

[21] Ignatov, M. et al. High accuracy prediction of PROTAC complex structures. *J. Am. Chem. Soc.* **145**(13), 7123–7135 (2023). 36961978 10.1021/jacs.2c09387 PMC10240388

[22] Ohue, M. et al. MEGADOCK 4.0: An ultra–high-performance protein–protein docking software for heterogeneous supercomputers. *Bioinformatics* **30**(22), 3281–3283 (2014). 25100686 10.1093/bioinformatics/btu532 PMC4221127

[23] Shimoda, T., Ishida, T., Suzuki, S., Ohue, M., & Akiyama, Y. Megadock-gpu: Acceleration of protein–protein docking calculation on gpus. In *Proceedings of the International Conference on Bioinformatics, Computational Biology and Biomedical Informatics* 883–889 (2013).

[24] Santos-Martins, D., Forli, S., Ramos, M. J. & Olson, A. J. AutoDock4Zn: An improved AutoDock force field for small-molecule docking to zinc metalloproteins. *J. Chem. Inf. Model.* **54**(8), 2371–2379 (2014). 24931227 10.1021/ci500209e PMC4144784

[25] Agrawal, P. et al. Benchmarking of different molecular docking methods for protein-peptide docking. *BMC Bioinform.* **19**, 105–124 (2019). 10.1186/s12859-018-2449-y PMC7394329 30717654

[26] Exner, T. E., Korb, O. & Ten Brink, T. New and improved features of the docking software PLANTS. *Chem. Central J.* **3**(Suppl-1), P16 (2009).

[27] Yang, J., Baek, M. & Seok, C. Galaxydock3: Protein-ligand docking that considers the full ligand conformational flexibility. *J. Comput. Chem.* **40**(31), 2739–2748 (2019). 31423613 10.1002/jcc.26050

[28] Lyskov, S. & Gray, J. J. The rosettadock server for local protein–protein docking. *Nucl. Acids Res.* **36**(suppl-2), W233–W238 (2008). 18442991 10.1093/nar/gkn216 PMC2447798

[29] Marze, N. A., Roy Burman, S. S., Sheffler, W. & Gray, J. J. Efficient flexible backbone protein–protein docking for challenging targets. *Bioinformatics* **34**(20), 3461–3469 (2018). 29718115 10.1093/bioinformatics/bty355 PMC6184633

[30] Varela, D., Karlin, V. & André, I. A memetic algorithm enables efficient local and global all-atom protein–protein docking with backbone and side-chain flexibility. *Structure* **30**(11), 1550–1558 (2022). 36265485 10.1016/j.str.2022.09.005

[31] Garzon, J. I. et al. Frodock: A new approach for fast rotational protein–protein docking. *Bioinformatics* **25**(19), 2544–2551 (2009). 19620099 10.1093/bioinformatics/btp447 PMC2800348

[32] Ugurlu, S. Y. et al. Cobdock: An accurate and practical machine learning-based consensus blind docking method. *J. Cheminform.* **16**(1), 5 (2024). 38212855 10.1186/s13321-023-00793-x PMC10785400

[33] Chen, R., Li, L. & Weng, Z. ZDOCK: An initial-stage protein-docking algorithm. *Proteins Struct. Funct. Bioinform.* **52**(1), 80–87 (2003). 10.1002/prot.10389 12784371

[34] Jiménez-García, B. et al. Lightdock: A new multi-scale approach to protein–protein docking. *Bioinformatics* **34**(1), 49–55 (2018). 28968719 10.1093/bioinformatics/btx555

[35] Ramírez-Aportela, E., López-Blanco, J. R. & Chacón, P. Frodock 2.0: Fast protein–protein docking server. *Bioinformatics* **32**(15), 2386–2388 (2016). 27153583 10.1093/bioinformatics/btw141

[36] Pierce, B. G. et al. ZDOCK server: Interactive docking prediction of protein–protein complexes and symmetric multimers. *Bioinformatics* **30**(12), 1771–1773 (2014). 24532726 10.1093/bioinformatics/btu097 PMC4058926

[37] Yuan, S., Chan, H. C. S. & Hu, Z. Using pymol as a platform for computational drug design. *Wiley Interdiscipl. Rev. Comput. Mol. Sci.* **7**(2), e1298 (2017).

[38] Wenfan, H. Rigid body protein docking by fast Fourier transform. In *Honours Year Project Report. School of Computing, National University of Singapore* (2005).

[39] Padhorny, D. et al. Protein–protein docking by fast generalized Fourier transforms on 5d rotational manifolds. *Proc. Natl. Acad. Sci.* **113**(30), E4286–E4293 (2016). 27412858 10.1073/pnas.1603929113 PMC4968711

[40] Padhorny, D. et al. Protein-ligand docking using FFT based sampling: D3r case study. *J. Comput.-Aided Mol. Des.* **32**, 225–230 (2018). 29101520 10.1007/s10822-017-0069-7 PMC5767528

[41] Pierce, B. G., Hourai, Y. & Weng, Z. Accelerating protein docking in ZDOCK using an advanced 3d convolution library. *PLoS ONE* **6**(9), e24657 (2011). 21949741 10.1371/journal.pone.0024657 PMC3176283

[42] Francoeur, P. G. et al. Three-dimensional convolutional neural networks and a cross-docked data set for structure-based drug design. *J. Chem. Inf. Model.* **60**(9), 4200–4215 (2020). 32865404 10.1021/acs.jcim.0c00411 PMC8902699

[43] Hou, T., Zhu, L., Chen, L. & Xiaojie, X. Mapping the binding site of a large set of quinazoline type EGF-R inhibitors using molecular field analyses and molecular docking studies. *J. Chem. Inf. Comput. Sci.* **43**(1), 273–287 (2003). 12546563 10.1021/ci025552a

[44] Van Zundert, G. C. P. et al. The DisVis and PowerFit web servers: Explorative and integrative modeling of biomolecular complexes. *J. Mol. Biol.* **429**(3), 399–407 (2017). 27939290 10.1016/j.jmb.2016.11.032

[45] Smith, G. R. & Sternberg, M. J. E. Prediction of protein–protein interactions by docking methods. *Current Opin. Struct. Biol.* **12**(1), 28–35 (2002). 10.1016/s0959-440x(02)00285-3 11839486

[46] Van Zundert, G. C. P. et al. The haddock2.2 web server: User-friendly integrative modeling of biomolecular complexes. *J. Mol. Biol.* **428**(4), 720–725 (2016). 26410586 10.1016/j.jmb.2015.09.014

[47] Schneidman-Duhovny, D., Inbar, Y., Nussinov, R. & Wolfson, H. J. PatchDock and SymmDock: Servers for rigid and symmetric docking. *Nucl. Acids Res.* **33**(suppl-2), W363–W367 (2005). 15980490 10.1093/nar/gki481 PMC1160241

[48] Pereira, G.P., Gouzien, C., Souza, P.C.T., & Martin, J. Challenges in predicting protac-mediated protein–protein interfaces with alphafold. *bioRxiv*, 2024–2003 (2024). 10.1093/bioadv/vbaf056 PMC11938821 40144455

[49] Basu, S. & Wallner, B. Dockq: A quality measure for protein–protein docking models. *PLoS ONE* **11**(8), e0161879 (2016). 27560519 10.1371/journal.pone.0161879 PMC4999177

[50] Ugurlu, S. Y., McDonald, D. & He, S. Mef-allosite: An accurate and robust multimodel ensemble feature selection for the allosteric site identification model. *J. Cheminform.* **16**, 116 (2024). 39444016 10.1186/s13321-024-00882-5 PMC11515501

[51] Ma, B., Liu, D., Wang, Z., Zhang, D., Jian, Y., Zhang, K., Zhou, T., Gao, Y., Fan, Y., Ma, J. et al. A top-down design approach for generating a peptide protac drug targeting androgen receptor for androgenetic alopecia therapy. *J. Med. Chem.* (2024). 10.1021/acs.jmedchem.4c00828 38836467

[52] Chen, T., Hong, L., Yudistyra, V., Vincoff, S., & Chatterjee, P. Generative design of therapeutics that bind and modulate protein states. *Current Opin. Biomed. Eng.* 100496 (2023).

[53] Sussman, J. L. et al. Protein data bank (PDB): Database of three-dimensional structural information of biological macromolecules. *Acta Crystallogr. Sect. D Biol. Crystallogr.* **54**(6), 1078–1084 (1998). 10089483 10.1107/s0907444998009378

[54] Weng, G. et al. Protac-db: An online database of protacs. *Nucl. Acids Res.* **49**(D1), D1381–D1387 (2021). 33010159 10.1093/nar/gkaa807 PMC7778940

[55] Ge, J., Li, S., Weng, G., Wang, H., Fang, M., Sun, H., Deng, Y., Hsieh, C.-Y., Li, D., & Hou, T. Protac-db 3.0: An updated database of protacs with extended pharmacokinetic parameters. *Nucl. Acids Res.*, gkae768 (2024). 10.1093/nar/gkae768 PMC11701630 39225044

