# Supplementary material for: MEGA PROTAC, MEGA DOCK-based PROTAC mediated ternary complex formation pipeline with sequential filtering and rank aggregation
Source: Sci Rep. 2025 Feb 14;15:5545. doi: 10.1038/s41598-024-83558-2 (PMC11829001; doi:10.1038/s41598-024-83558-2)
Supplement: Supplementary file 1 — Supplementary Information. [file 41598_2024_83558_MOESM1_ESM.pdf]

1 Supplementary Information for MEGA PROTAC: MEGADOCK-based PROTAC-Mediated Ternary Complex Formation Pipeline with Sequential Filtering integrated with Rank Aggregation

Supplementary information has been provided in addition to the main debate discussed earlier. The supplementary information expands the range and intricacy of our debate, providing a deeper understanding of the issue. Therefore, nine main titles have been used in supplementary information: (i) Summary of MEGA PROTAC protocol, (ii) Cavity detection tool for PROTAC protocol, (iii) Background of filtration criteria, (iv) Fundamentals of Rank Aggregation, (v) Fundamentals of Clustering, (vi) The Background about additional performance evaluation metrics, (vii) Comparison Analysis: Molecular Dynamic Simulation (MD) for BOTCP vs MEGA PROTAC, and (viii) Examining Ternary Structure Prediction for Methods via Visual Analysis and (ix) MEGADOCK Parameters. With the help of these five supplementary pieces of knowledge, MEGA PROTAC's performance and nature can be deeply understood.

1.1 Summary of MEGA PROTAC protocol

MEGA PROTAC employs the MEGADOCK discoveries as pre-grid refinement candidate protein-protein complexes (PPCs) to establish an initial exploration area for PPCs. The 5000 PPCs produced by MEGADOCK have been used as an initial exploration area. Then, a sequential filtration strategy combined with rank aggregation was employed to choose a subset of promising PPCs for ternary structures. Once a subset (200 candidates) is selected, MEGA PROTAC uses a grid search method focusing on translation and rotation. MEGA PROTAC created an exploration area of 68800 PPCs using the subset in the translation grid search. Then, MEGA PROAC filtered the unpromising structures and selected the top 200 translated PPCs. The top 200 translated PPCs have produced 68800 PPCs in a rotational grid search. After filtering out unpromising rotated PPCs, the rest of the PPCs were clustered, and clusters were filtered based on whether proteins with low energy scores existed in the cluster. Finally, the unfiltered PPCs have been re-clustered and ordered using our rank aggregation approach.

1.2 Cavity detection tool for PROTAC protocol

A trade-off exists between the performance of molecular docking systems and their time efficiency in PROTAC screening. Increased performance necessitates greater flexibility, resulting in a longer execution time for the program; for example, the inclusion of FRODOCK [1] and RosettaDock [2] has been observed to significantly increase the runtime of the approach [3, 4]. However, in PROTAC screening, five components must be optimised: two proteins, an anchor, a warhead, and a linker. In comparison, conventional virtual screening only focuses on two components, namely the target and the ligand. Therefore, it is essential to note that the PROTAC screening space is considerably larger than that of conventional virtual screening studies. Programs, such as FRODOCK [1] and RosettaDock [2], require much time and computational resources

for PROTAC screening. Therefore, selecting a molecular docking program is a crucial aspect of the investigation, as it plays a pivotal role in optimising time efficiency. An investigation into the already available molecular docking programs was conducted to enhance the efficiency of our procedure by augmenting its performance. (Table 1).

Table 1 The summary feature and groups of molecular docking programs in the literature. The categorisation of molecular docking can be based on two factors: the type of docking and the type of input. Docking types can be classified into two categories: (i) local docking and (ii) global (blind) docking. The process of local docking involves the execution of docking algorithms on a designated and predetermined position of the target protein. In cases where the binding site's precise position is unknown, it becomes necessary for molecular docking systems to do a comprehensive search of the complete protein structure to identify potential binding sites and subsequently execute the docking process. Such a process is called global (blind) docking. Additionally, it is possible to categorise molecular docking programs into three distinct classes according to their inputs: (i) small molecule-protein docking programs, (ii) peptide-protein docking programs, (iii) protein-protein, and (iv) Nucleic acid-protein docking programs. The molecular docking programs have undergone testing to determine their capability to execute ternary docking. Ternary docking represents the ability to perform docking for two proteins and one ligand. Finally, the table demonstrates the features of docking programs in the literature.

**Docking Program**	**Docking type**	**Input type**	**Ternary Docking**	**Features**
Vina	Local docking	Small molecule-protein	No	High performance: 81% accuracy [5]<br>Ease of use<br>Common<br>A high number of different pose locations
PLANTS	Local docking	Small molecule-protein	No	High performance: 87% [6, 7]<br>Relatively fast<br>A high number of different pose locations
GalaxyDock3	Local docking	Small molecule-protein	No	High performance [8]<br>Ease of use<br>A high number of different pose locations<br>Full Ligand Conformational Flexibility [8]
Rosetta	Local docking	Protein-protein	**Yes**	High performance [2, 9]<br>The high number of independent structures45<br>Moderate or low speed [10]
FRODOCK 2.0	Local docking	Protein-protein	**Yes**	Extra knowledge-based potential [6]<br>High Performance [6, 1]
CoBDock	Global (blind) docking	Small molecule-protein	**No**	High performance [11]<br>High automation [11]
MEGADOCK 4.0	Global (blind) docking	Protein-protein	**Yes**	High speed [12, 13]<br>Relatively high-performance43
ZDOCK	Global (blind) docking	Peptide-protein, protein-protein	**Yes**	High performance: 85.71% [14]<br>Blind (Global) docking<br>A high number of poses in similar locations
LightDock	Global (blind) docking	Protein-protein, peptide-protein, DNA-protein	No	Conformational flexibility [15]<br>A variety of scoring functions44

In previous research on developing ternary structures, molecular docking has been employed to investigate proteins that possess ligands. Therefore, an assessment has been conducted on each molecular docking program included in Table 1 to determine its capability to conduct protein-protein docking in the presence of ligands. The

"ternary docking" characteristic in the table illustrates the ability of programs to perform docking successfully, even in cases where at least one of the proteins possesses a ligand, like anchor and warhead. After the elimination of molecular docking programs based on the "ternary docking" feature, only four programs, MEGADOCK 4.0 [12], FRODOCK [1], ZDOCK [14], and RosettaDock [2], remained for protein-protein docking step with anchor and warhead. Hence, only four molecular docking programs were considered for subsequent evaluation.

The primary docking software: MEGADOCK

MEGADOCK employs a Katchalski-Katzir algorithm, specifically a conventional Fast Fourier Transform (FFT)-based rigid-docking approach [12]. The scoring function of the original model is determined by a single correlation function, which takes into account shape complementarity, electrostatics, and desolvation-free energy. Using numerous correlation functions and conducting several FFT calculations allows for faster calculation, favourably compared to other methods that assess many impacts [12]. Also, the software, MEGADOCK 4.0, is implemented using a combination of hybrid CUDA, MPI, and OpenMP parallelisation techniques. Minimising memory utilisation is crucial in systems with numerous CPU cores, multiple GPUs per node, and limited memory capacity (e.g., a mere 6 GB on an NVIDIA Tesla K20X GPU). They allocated a single docking task to each node and subsequently distributed the computation of ligand rotation using thread parallelisation using both CPU cores and GPUs [12].

MEGADOCK offers distinct parameters for regulating docking, including the ability to adjust MEGADOCK-grid size and penalty scores. Increasing the grid size parameter of MEGADOCK generates pre-grid refinement candidate PPCs in a short period while also ensuring a high level of diversity [12, 16]. Also, the remaining two fundamental penalty score parameters can enhance identifying a broader range of protein complexes by regulating the spatial separation between two input proteins [12, 16]. These features help to make the observed protein-protein complexes more diverse, which makes it less likely that the “true ternary” structure will be missed. A high level of diversity is beneficial in the first stages of the process, as it can lead to the generation of acceptable structures. Once these acceptable structures are successfully chosen, they can enhance the performance of MEGA PROTAC.

Consequently, MEGADOCK can increase diversity by incorporating factors such as MEGADOCK-grid size and penalty scores. The filtration process allows us to choose possible locations from various candidate PPCs. Rank aggregation is a method used to determine the most promising candidates by ordering them. Hence, due to the aforementioned benefits of MEGADOCK, MEGADOCK surpasses RosettaDock and FRODOCK as the primary molecular docking software. Therefore, the MEGADOCK software has been selected as the main docking method for our investigation."

1.3 Background of filtration criteria

***MDAnalysis score, stability-based filtration:*.** MDAnalysis is a Python module specifically developed to analyse molecular dynamics trajectories and atomic simulation data [17]. The module provides a wide range of tools that facilitate the parsing,

manipulation, and analysis of molecular structures. This feature renders it highly valuable for investigating protein structures obtained through molecular dynamics simulations or experimental data.

Within the framework of the function that filters unpromising structures, the term "norms" pertains to the Euclidean norms associated with the position vectors of the C± atoms within a protein. The aforementioned norms serve as indicators of the magnitudes of these vectors, so offering insights into the spatial arrangement of the atoms inside the protein architecture. The function computes a quality score that indicates the protein molecule's overall spatial organisation and stability by computing the mean of these norms. This score can be a quantitative indicator of protein quality, which is valuable for conducting comparative analyses or investigations based on protein structure [17].

SASA-based filtration:. Following stability-based filtering, Filtering based on the solvent-accessible surface area (SASA) of proteins, which has historically been regarded as a critical variable in protein folding and stability investigations, is employed. SASA provides more structural information about structure; for example, the highest values of SASA represent the most open binding site, while the lowest values represent the most closed pocket, which helps predict the dynamic behaviour of the binding site. SASA also can adapt ligands to conform to different sizes and shapes [18]. SASA has been utilised in many applications, including determining protein structure, protein-ligand docking, and analysing protein-protein interactions [19]. As a comprehensive structure representation, SASA provides information about stability, conservability of binding sites, and molecular interactions for complex protein structures. Therefore, it serves as a robust filter to eliminate unpromising PPCs. For instance, a PPC with a lower SASA suggests a smaller distance between the proteins. In contrast, a PROTAC molecule is too large to fit in such complexes. Therefore, SASA can function as an effective protein representation to improve performance in PROTAC screening due to its thorough structure depiction. Consequently, SASA features are designed to be part of MEGA PROTAC's pipeline to improve the performance of the allosteric binding site using FreeSASA [19].

A strong link exists between SASA and distances between proteins since a higher SASA value means a larger surface area where solvents/ligands reach the protein's surface. PRosettaC demonstrated that a potentially advantageous ternary structure complex might consist of 20 Å across proteins in a ternary structure. Since PROTAC structures are much larger than small molecules, the most effective filtration approach to maintain a larger spacing between proteins would be to use SASA. Consequently, the protein's lower half, determined by its total SASA value, has been eliminated to expedite the process and eliminate proteins having more conservative areas. Only proteins with a larger SASA were retained for subsequent filtration, saving time and funds.

Energy-based filtration:. OpenBabel is an open chemical toolbox with several tools describing chemicals, including Obenergy [20]. Obenergy calculates the energies of a structure by using three different force fields: (i) Universal Force Field (UFF), (ii) General AMBER Force Field (Gaff) and (iii) Gchemical. (i) UFF can replicate most structural traits in the periodic table. All elements may have their geometry

optimised by this force field, which works well with inorganic and organometallic compounds. (ii) The general AMBER force field was designed mainly for biomolecules, such as proteins, DNA, RNA, and carbohydrates. (iii) Ghemical provides a force field for geometry optimisation and molecular dynamics. These force fields provide several energy features, such as stretching, angle bending, and torsional energy [20].

Following BOTCP [21], Obenergy UFF-based filter criteria were adopted for the remaining PPCs. Unlike Weng et al. [3], who used GAFF for calculating the overall energy of protein complexes, UFF is employed by us because of its superior speed compared to GAFF and its capability to specify a more comprehensive range of elements (e.g., Platinum). Therefore, UFF allows for the unrestricted design of PROTAC, even if the warhead has an uncommon element, such as platinum. For example, cisplatin, an anticancer medicine containing platinum, is known for the presence of metal ions in certain medications. UFF can outperform GAFF, particularly when such cisplatin assumes the role of a warhead. In addition, BOTCP [21] utilised the UFF in their research and achieved better results than Weng et al. [3]. Consequently, the utilisation of UFF has been implemented in our protocol as opposed to GAFF, following the BOTCP study [21].

The fixed threshold used in Weng et al.'s study [3] may lead to data loss, such as when a large protein (> 20,000 atoms). Consequently, a rudimentary dynamic threshold relies on input energies to preserve crucial data to the greatest extent possible, drawing inspiration from phenomena in reaction energy states. Intermediate structures of ternary structures may have higher or lower than the initial total energy. Therefore, the total energy of the input proteins is multiplied by 2 and divided by 2 to find lower and higher energy thresholds in kJ/mol. The total energy of PPC between thresholds has been kept for further filtration, while the rest have been eliminated. Consequently, the pipeline became more robust against input features like protein size or atom number.

Protein Interaction Z-score quality-based filtration:. It is expected to take into account the geometric and chemical complementarity of the interactors to propose potential protein-protein interactions (PPIs) [22]. The challenge usually entails selecting several conformations of the interactors about each other and subsequently assigning scores to each of these putative relationships. The Protein Interaction Z-Score Assessment (PIZSA) method leverages the observation that high observed/-expected ratios suggest favourable energetics [23]. PIZSA specifically utilises pairwise connections of amino acids that are in close physical proximity across the protein-protein interaction (PPI) interface [68] to calculate Z-score and stability classification [23]. The Z-score measures PCCs' stability, making it ideal for removing particularly unstable protein complexes. Also, PIZSA classifies proteins according to whether their structure is stable by using a unique threshold for each protein. Thus, the Z-Score and stability categorization of PIZSA are the exclusive characteristics used to assess protein structures.

PIZSA calculates a Z-score and assesses the stability of PPIs. The Z-score cutoff was optimised to 1.5 in the original PIZSA study [23]. A lower Z-score of 0.5 was employed to eliminate unpromising MEGADOCK pre-grid refinement candidate PPCs

without losing promising structures. Furthermore, the PIZSA-based stability classification has not been utilised for MEGADOCK pre-grid refinement candidate PPCs to retain the maximum number of pre-grid refinement candidate PPCs. By employing a higher Z-score threshold and doing stability evaluations, nearly all MEGADOCK pre-grid refinement candidate PPCs have been filtered out for 6HAY-BA. Consequently, the MEGADOCK 5,000 PCC results have employed lenient criteria to maintain a sufficiently large space for a grid search.

As for grid search parameters, the higher Z-score threshold (1.0) and stability classification assessment have been considered to eliminate mostly unpromising protein structures. Grid search starters from 68,800 PPCs, which is almost 14 times larger than MEGADOCK pre-grid refinement candidate PPCs numbers. Therefore, even if 1 for Z-score threshold and stability classification assessment is used, 7,000-8,000 protein complexes have been kept for PIZSA stability analysis. After PIZSA stability-based filtration, around 3,000 complexes remained for VoroMQA analysis. Consequently, the stricter threshold of PIZSA and stability-based filtration were utilised in the grid search before VoroMQA.

VoroMQA-based quality assessment for rank aggregation:. VoroMQA is a novel approach to estimating protein structure quality using interatomic contact areas. The VoroMQA integrates the concept of statistical potentials with utilising inter-atomic contact regions as an alternative to geographical distances. Contact areas are utilised to describe and integrate explicit interactions between protein atoms and implicit interactions between protein atoms and solvent. These contact areas are obtained by applying Voronoi tessellation of protein structure. VoroMQA generates scores within a predetermined range of 0 to 1 at the atomic, residue, and global levels.

As in the study by Weng et al. [3], VoroMQA has been used in rank aggregation to order proteins. Following Weng et al. [3], MEGA PROATC utilises VoroMQA in rank aggregation instead of filtration. Since the protein with the lowest VoroMQA score will be positioned at the bottom of the rankings, these proteins should be eliminated by picking the top 200 promising PPCs or ignored in practical usage. Consequently, the utilisation of VoroMQA solely for rank aggregation results in time savings by preventing the need for redundancy filtration based on VoroMQA.

1.4 Fundamentals of Rank Aggregation

There are four primary justifications for the need for ranking: (i) prioritizing candidate complexes, (ii) efficient exploration vs. exploitation, (iii) limited resources, and (iv) data analysis and Interpretation [24]. (i) A method investigates a wide range of possible arrangements for the ternary complex consisting of a protein, a PROTAC molecule, and an E3 ubiquitin ligase. Exploring each individual arrangement would entail significant processing costs and consume a considerable amount of time. Ranking is a method used to prioritise the most favourable configurations for review. The prioritisation is determined by a score function that assesses the stability or appropriateness of the complex. (ii) The objective of the method is to determine the most optimal arrangement for the ternary complex. Ranking provides a means to balance exploration, which involves trying out various configurations, and exploitation, which

involves focusing on promising areas. At first, discovery may hold greater significance in uncovering novel opportunities. Ranking might subsequently direct a method towards configurations that exhibit more anticipated stability, drawing on previous assessments. (iii) There could be constraints on the computational resources or time allocated for grid search. Ranking facilitates the effective allocation of resources by prioritising configurations that are ranked highest and have the greatest potential for success. (iv) Following the grid search, a method produces data regarding the investigated configurations. Ranking facilitates the identification of the most highly ranked complexes, which may then be subjected to further examination and analysis. This enables researchers to concentrate on the most auspicious candidates for experimental verification [24, 25].

1.5 Fundamentals of Clustering

Clustering is necessary for the method design for PROTAC screening because of four main reasons: (i) improved exploration strategy, (ii) data augmentation and knowledge transfer, (iii) identifying diverse candidates, and (iv) data visualization and analysis. (i) The potential range of ternary complex configurations in a given approach can be extensive. Clustering facilitates the grouping of similar arrangements. This enables the approach to investigate a broader range of places inside this space efficiently. The technique can concentrate its evaluations on locations with a higher probability of success by identifying clusters of promising configurations. (ii) Throughout the optimisation process, a technique gathers information on the configurations that have been examined and their projected levels of stability. Grouping similar settings together enables learning from this data more efficiently. The insights obtained from assessing a single configuration within a cluster can be utilised to enhance the forecasts for other configurations. This approach can be advantageous when working with a restricted amount of data since it enables the application of knowledge acquired from one section of the search domain to other interconnected regions. (iii) Although it is crucial to prioritise high-scoring configurations, it can also be advantageous to have some variety in the configurations that are examined. Clustering can mitigate the risk of a method becoming trapped in a local optimum. By incorporating configurations from several clusters, regardless of their individual rankings, a method can systematically investigate a wider spectrum of options and potentially uncover superior answers. (iv) Clustering the examined setups might be a beneficial tool for researchers. By visualising these clusters, researchers can obtain insights into the entire search area and detect potential patterns or connections among different configurations. This can facilitate interpreting the outcomes obtained from a technique and potentially provide guidance for future enhancements. Therefore, MEGA PROTAC applied a clustering approach like previous studies [21].

1.6 The Background about additional performance evaluation metrics

DockQ is a widely utilised scoring function in protein-protein docking simulations. The objective is to determine the binding affinity or stability of a complex formed by two

molecules, such as a protein and a small molecule. DockQ is used to evaluate the anticipated stability of ternary complexes (consisting of a protein, PROTAC molecule, and E3 ubiquitin ligase). Overall, the DockQ score is a composite statistic that likely integrates f(nat), I_RMSD, and L_RMSD metrics to provide a comprehensive assessment of the expected stability of the ternary complex [26].

The assessment of model quality was conducted using the criteria outlined in the Critical Assessment of Protein Structure Prediction (CAPRI) [27], which encompassed three primary parameters: f(nat), I_RMSD, and L_RMSD as a component of DockQ score [26].

F(nat) is defined as the proportion of native interfacial connections that are maintained in the interface of the projected complex [26]. The variable f(nat) denotes the proportion of native contacts inside the target that are successfully retrieved by the model. Clashes are defined as atomic contacts that occur at distances below 3 Å. Predictions that exhibit an excessive number of clashes are deemed ineligible for consideration [27].

L_RMSD is the root mean square deviation (RMSD) of the backbone over the shared set of ligand residues following the superimposition of receptor proteins [27]. Similarly, I_RMSD represents the backbone RMSD computed over the standard set of interface residues subsequent to the structural alignment of these residues. The term "interface residue" is used to describe a residue in a molecular system where any of its atoms, except hydrogen atoms, are found within a distance of 10 Å from any of the atoms belonging to the binding partner. Based on the values of these three-parameter models, they are classified into four distinct categories of quality scores, namely high quality, medium quality, acceptable quality, and inaccurate [27].

1.7 Comparison Analysis: Molecular Dynamic Simulation (MD) for BOTCP vs MEGA PROTAC

Molecular dynamic (MD) simulations significantly provide better results than molecular docking [28, 29]. Therefore, molecular dynamic simulations were used by BOTCP (MD) in their refinement step to improve their results. Understanding the strengths and weaknesses of MEGA PROTAC, the application of molecular dynamic simulations in BOTCP (MD) results was compared with our grid search results. The methods have been evaluated based on (i) Quality assessment using DockQ score and (ii) Ranking performance assessment. Finally, the limitations of BOTCP have been discussed in the "Limitations of BOTCP" section.

1.7.1 Quality assessment using DockQ score

The quality evaluation approaches, namely DockQ, were utilised to evaluate the performance of the methodologies. The BOTCP (MD) system offered four improved quality classifications based on the DockQ score, namely 5T35-DA, 6HAX-FE, 6HAY-FE, and 7Q2J-CD (Table 2). MEGA PROTAC outperforms in eight out of the 22 test sets, namely 6BN7-BC, 6BOY-BC, 6HR2-BA, 6HR2-FE, 6W7O-CA, 6W8I-DA, 6W8I-FC, and 7KHH-CD, providing superior classification quality (Table 2). The quality classification for the remaining proteins was consistent across all approaches.

MEGA PROTAC exhibited a 36.36% improvement in classification performance. However, BOTCP (MD) demonstrated an 18.182% of all cases superior classification performance compared to MEGA PROTAC. Therefore, the figure illustrates that MEGA PROTAC exhibits double the quality classification compared to the molecular dynamic simulation of BOTCP (MD). The low performance may be attributed to the constrained efficacy of the pre-refinement and the insufficient allocation of time for MD towards achieving superior outcomes. The suboptimal performance of pre-refinement processes may be attributed to the restricted effectiveness of the BOTCP (MD) filtering strategy, which hinders the achievement of better outcomes. Also, the higher median to alter the classification of structure quality for MEGA PROTAC supports that MEGA PROTAC is likely to provide better-qualified structures than BOTCP (MD).

DockQ scores provide a comprehensive assessment of the overall quality performance of each model. The MD simulation performed by BOTCP (MD) resulted in a higher DockQ score of 9 out of 22. The improved DockQ score ranged from 0.07 to 0.491 (Table 2). The largest disparity in DockQ scores is reported for the protein complex 7Q2J-CD. The 7Q2J-CD protein obtained a DockQ value of 0.851 via BOTCP (MD), while the best ternary structure predicted by MEGA PROTAC had a DockQ score of 0.36. MEGA PROTAC achieved a higher DockQ score for 12 tests out of a total of 22, without the need for further steps to improve performance. The MEGA PROTAC exhibits a higher DockQ score of 0.01 to 0.354 for those 12 test cases. The largest increase in DockQ score has been observed for the protein 7KHH-CD. The BOTCP (MD) algorithm yielded a DockQ value of 0.402 for the protein structure of 7KHH-CD, while the MEGA PROTAC algorithm produced a DockQ score of 0.756 for the same protein structure. The statistics illustrate that MEGA PROTAC, despite its absence of time-consuming processes and flexibility considerations, delivered a 33% higher DockQ score than BOTCP (MD), with MEGA PROTAC providing a higher DockQ score 12 times compared to BOTCP (MD)'s 9 times.

The mean and median DockQ scores are vital to demonstrating the overall quality performance of MEGA PROTAC and BOTCP (MD) (Table 2). BOTCP (MD) increased the mean and median of DockQ score using molecular dynamic simulation from 0.467 and 0.420 (Main text) to 0.548 and 0.450 (Table 2). On the other hand, MEGA PROTAC provided 0.554 for the mean and 0.568 for the mean DockQ score values. Although there is no significant improvement in the mean DockQ score, the figures demonstrated that MEGA PROTAC enhanced 26.222% of the median. The improvement in median without any retirement application for MEGA PROTAC demonstrates that MEGA PROTAC has great potential to be investigated and improved for PROTAC screening.

1.7.2 Ranking performance assessment

There are two approaches to ranking and evaluating the performance of methods: (i) ranking the performance based on the most qualified structure and (ii) ranking the performance based on the first acceptable structures. (i) Table 4 demonstrates the ranking performance for the cluster having the highest DockQ score. (ii) Table 3 indicates the ranking performance for the cluster having the first acceptable structure

Table 2 The table presents the top DockQ scores for 22 test cases obtained by BOTCP (MD) and MEGA PROTAC. In addition, it presents f(nat), I-RMSD, and L-RMSD values, which act as indications of the accuracy of the methods' predictions. The final number indicates the greatest attainable DockQ score computed in BOTCP (MD), representing the maximum achievable DockQ score [21].

PDB ID	BOTCP (MD)					MEGA PROTAC					Max DockQ
	f(nat)	I-RMSD	L-RMSD	DockQ	Class	f(nat)	I-RMSD	L-RMSD	DockQ	Class	
5T35-DA	**1**	**0.997**	**3.561**	**0.848**	**H**	**1**	1.361	4.46	0.778	M	0.85
5T35-HE	0.75	**1.302**	**2.909**	0.738	M	**1**	1.392	4	**0.785**	**M**	0.88
6BN7-BC	0.333	**3.172**	**5.593**	0.405	A	**1**	3.543	7.102	**0.58**	**M**	0.8
6BOY-BC	0.622	3.33	10.746	0.392	A	**0.9**	**2.926**	**6.111**	**0.589**	**M**	0.81
6HAX-BA	0.474	**1.316**	**4.208**	**0.614**	**M**	**1**	2.486	11.479	0.54	**M**	0.85
6HAX-FE	0.421	**1.484**	**4.353**	**0.573**	**M**	**0.667**	6.787	17.335	0.302	A	0.85
6HAY-BA	0.812	**1.507**	**5.509**	**0.671**	**M**	**1**	4.216	7.585	0.556	**M**	0.89
6HAY-FE	**0.875**	**1.772**	**6.879**	**0.632**	**M**	0.333	4.234	12.669	0.252	A	0.87
6HR2-BA	0.519	**2.046**	10.364	0.423	A	**1**	4.317	**10.194**	**0.506**	**M**	0.81
6HR2-FE	0.481	**2.213**	11.54	0.383	A	**1**	4.393	**10.415**	**0.501**	**M**	0.84
6SIS-DA	**0.818**	**1.067**	**4.217**	**0.762**	**M**	0.8	1.403	6.97	0.644	**M**	0.86
6SIS-HE	0.909	**1.134**	**4.097**	**0.786**	**M**	**1**	1.509	4.727	0.754	**M**	0.83
6W7O-CA	**1**	3.492	17.512	0.449	A	**1**	**2.957**	**5.682**	**0.632**	**M**	0.84
6W7O-DB	**1**	**3.475**	17.447	**0.45**	**A**	0.818	3.626	**11.341**	0.441	**A**	0.83
6W8I-DA	0.273	2.641	7.305	0.364	A	**1**	**1.894**	**5.359**	**0.7**	**M**	0.84
6W8I-EB	0.323	2.794	**5.341**	0.421	**A**	**0.625**	**2.281**	7.857	**0.489**	**A**	0.79
6W8I-FC	0.875	4.298	16.408	0.398	A	**1**	**1.931**	**9.789**	**0.602**	**M**	0.86
6ZHC-AD	0.235	2.718	**4.896**	0.407	**A**	**1**	**3.766**	23.586	**0.417**	**A**	0.91
7JTO-LB	-	-	-	-	-	**0.286**	**2.287**	**9.741**	**0.34**	**A**	0.76
7JTP-LA	0.684	2.424	**5.75**	0.549	**M**	**1**	**1.856**	6.57	**0.674**	**M**	0.84
7KHH-CD	0.429	2.967	7.303	0.402	A	**0.8**	**1.284**	**2.974**	**0.756**	**M**	0.92
7Q2J-CD	**0.9**	**0.926**	**2.373**	**0.851**	**H**	0.5	3.694	9.617	0.36	A	0.88
Mean	0.654	**2.242**	**7.539**	0.548	**M**	**0.851**	2.916	8.889	**0.554**	**M**	0.846
Median	0.684	**2.213**	**5.593**	0.45	A	**1**	2.706	7.721	**0.568**	**M**	0.845

(>= DockQ score, 0.23). The tables 4 and 3 demonstrate that the complete cluster numbers for MEGA PROTAC and BOTCP are demonstrated to do a thorough comparison analysis.

Table 3 The table displays the performance rankings for clusters that possess a DockQ score of at least 0.23, which is considered acceptable. Regardless of individual rankings, the mean and median have been calculated to provide a comprehensive view of the overall rating. The table displays the hierarchical ordering of MEGA PROTAC and BOTCP clusters. In addition, the total number of clusters has been depicted. Unfortunately, the proportion of near-native representation for BOTCP (MD) has not been published. Hence, this statistic has been disregarded in this context.

**PDB ID**	**BOTCP (MD)**		**MEGA PROTAC**	
	First Acc. Cluster Rank	Total Cluster Number	First Acc. Cluster Num.	Total Cluster Number
5T35-DA	**1**	89	2	**59**
5T35-HE	**1**	89	7	**66**
6BN7-BC	8	130	**1**	**70**
6BOY-BC	11	133	**3**	**93**
6HAX-BA	**1**	129	7	**85**
6HAX-FE	**1**	129	3	**107**
6HAY-BA	**1**	117	18	**94**
6HAY-FE	**1**	117	19	**85**
6HR2-BA	8	92	**1**	**90**
6HR2-FE	8	92	**5**	**90**
6SIS-DA	**2**	**70**	4	72
6SIS-HE	**2**	70	7	**60**
6W7O-CA	42	99	**4**	**92**
6W7O-DB	42	99	**2**	**62**
6W8I-DA	10	113	**5**	**82**
6W8I-EB	86	128	**2**	**60**
6W8I-FC	21	113	**1**	**65**
6ZHC-AD	None	None	**14**	**54**
7JTO-LB	13	None	**1**	**75**
7JTP-LA	7	**46**	**1**	97
7KHH-CD	28	121	**1**	**65**
7Q2J-CD	6	**82**	**2**	93
**Mean**	14.286	102.9	**5**	**78**
**Median**	8	106	**3**	**78.5**

Applying molecular dynamic simulation of BOTCP (MD) significantly decreases about half of their total groups (Tables 4 and 3). More precisely, BOTCP (MD) had a mean of 102.9 and a mean of 106 for the total cluster number. Nevertheless, MEGA PROTAC exhibits lesser cluster quantities than BOTCP (MD), even without any structural modification, with a mean of 78 and a median of 78.5. Based on the cluster numbers, MEGA PROTAC identified approximately 20% more concentrated protein structures compared to BOTCP (MD).

As for the percentage of near-native conformations (Table 4), BOTCP (MD) has provided higher percentages than MEGA PROTAC. MEGA PROTAC has a mean

Table 4 The table presents the performance rankings for clusters that include the protein with the highest DockQ score. Irrespective of individual rankings, the mean and median have been computed to offer a basic understanding of the overall rating. The table presents the cluster ranking for MEGA PROTAC and BOTCP (MD). Furthermore, the overall number of clusters has been illustrated. Ultimately, the near-native percentage indicates the ratio of satisfactory protein within that particular cluster.

PDB ID	BOTCP (MD)			MEGA PROTAC		
	Cluster rank	Total Cluster Number	% Near-native	Cluster rank	Total Cluster Number	% Near-native
5T35-DA	11	89	**100**	**7**	**59**	71.818
5T35-HE	32	89	**100**	**13**	**66**	37.500
6BN7-BC	19	130	30	**2**	**70**	**74.282**
6BOY-BC	10	133	83	**4**	**93**	**97.143**
6HAX-BA	9	129	**100**	**8**	**85**	82.051
6HAX-FE	9	129	**100**	**3**	**107**	32.099
6HAY-BA	42	117	**100**	**36**	**94**	**100**
6HAY-FE	**42**	117	**100**	60	**85**	14.286
6HR2-BA	33	92	**100**	**6**	**90**	12.121
6HR2-FE	**33**	92	**100**	35	**90**	26.667
6SIS-DA	**3**	**70**	**94**	4	72	53.333
6SIS-HE	**3**	70	**96.5**	17	**60**	55.844
6W7O-CA	**4**	99	40	62	**92**	**100**
6W7O-DB	**4**	99	40	58	**62**	**100**
6W8I-DA	**3**	113	**100**	11	**82**	66.667
6W8I-EB	39	128	**100**	**32**	**60**	16.667
6W8I-FC	4	113	**100**	**1**	**65**	52.381
6ZHC-AD	**10**	None	None	29	**54**	**90.909**
7JTO-LB	None	None	None	**27**	**75**	**31.818**
7JTP-LA	**1**	**46**	15.8	4	97	**74.257**
7KHH-CD	25	121	66.7	**12**	**65**	**65.274**
7Q2J-CD	**2**	**82**	**100**	14	93	11.429
**Mean**	**16.095**	102.900	**83.3**	20.227	**78**	57.570
**Median**	**10**	106.000	**100**	12.5	**78.5**	60.559

and median value of approximately 60, but BOTCP (MD) has a considerably higher mean and median value of nearly 90. Regrettably, BOTCP (MD) cannot be utilised to re-execute and examine the cause behind MEGA PROTAC's constraint to enhance it. Nevertheless, BOTCP (MD) achieved better results than MEGA PROTAC in terms of the percentage of near-native.

Discovering suitable structures among the better ranks greatly enhances the practical applicability of approaches. Therefore, there are two assessments for programs: (i) ranking performance for the cluster having the highest DockQ score (Table 4), and (ii) ranking performance for the cluster having the first acceptable DockQ score (Table 3). The integration of these two performances indicates the overall ranking of MEGA PROTAC and BOTCP (MD).

Table 4 demonstrates the ranking performance for the cluster having the highest DockQ score. MEGA PROTAC outperformed BOTCP (MD) in 12 out of 22 test instances, resulting in a higher rating of 54.545% based on ranking performance (Table 2). The overall lower mean and median values of ranks for BOTCP (MD) (Table 4) indicate that the BOTCP (MD) ranking has the potential to outperform

MEGA PROTAC. They increased their ranking power by filtering pre-refinement structures and using them in molecular dynamic simulation steps to create more homogenous and qualified structures. Nevertheless, the MEGA PROTAC, although a simple approach, exhibited significantly considerable ranking performance for clusters having the highest DockQ score compared to BOTCP (MD).

Table 3 indicates the ranking performance for the cluster having the first acceptable DockQ score. The ranking for the cluster with the first acceptable DockQ score directly indicates the practical usage impact of MEGA PROTAC and BOTCP (MD). MEGA PROTAC demonstrated superior performance compared to BOTCP (MD) in 13 out of 22 test instances, leading to a higher grade of 59.090% based on ranking performance (Table 4). The mean and median rankings for BOTCP (MD) were 14.286 and 8.000, respectively, while MEGA PROTAC outperformed BOTCP (MD) with a mean score of 5 and a median ranking of 3. MEGA PROTAC significantly enhanced both the mean and median by nearly three times. Consequently, MEGA PROTAC outperformed BOTCP (MD) in terms of ranking performance.

1.7.3 The practical usage impact of programs: BOTCP (MD) and MEGA PROTAC

BOTCP (MD) assigned the first structure a cluster rank greater than 40 for 3 out of 22 (13.636%) protein complexes: 6W7O-CA, 6W7O-DB, and 6W8I-EB (Table 3 and Figure 1). Unfortunately, such an unpromising rank requires time-consuming manual filtration in the research of PROTAC design, or researchers may overlook acceptable structures for three proteins. Conversely, MEGA PROTAC achieved a ranking performance that was either lower or equal to 20 across all test cases, demonstrating its strong and reliable performance in ranking. The approximately three times lower mean and median values further substantiate that the MEGA PROTAC ranking performance surpasses that of BOTCP (MD), even considering the impact of clustering filtration and molecular dynamic simulation stages on their respective ranking performances.

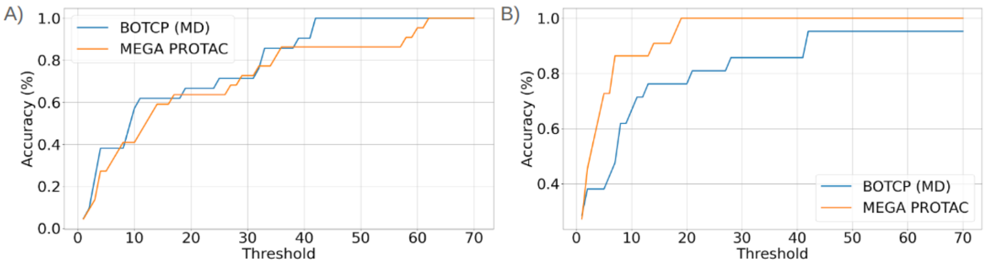

Fig. 1 The figure illustrates the accuracy of two methods, BOTCP (MD) and MEGA PROTAC, at various thresholds. Each threshold corresponds to a ranking value, and any value lower than the threshold is considered correct. For a given threshold, any rank that is less than or equal to the threshold is considered correct. The number of correct cases is then divided by the total number of tests, which is 22, to calculate the accuracy. "A" represents the accuracy performance for the cluster with the highest DockQ score, while "B" represents the ranking accuracy for the cluster with at least one acceptable PPC (>= 0.23 DockQ score).

Figure 1 demonstrates the accuracy of BOTCP (MD) and MEGA PROTAC for every threshold. Figure 1 A suggests that BOTCP (MD) in blue has better ranking performance for clusters having the highest DockQ score than MEGA PROTAC in yellow. On the other hand, finding a cluster having at least one acceptable PPC directly shows the impact of methods on real-life studies. Figure 1 B, demonstrating the ranking for clusters having at least one acceptable PPC, indicates that MEGA PROTAC in yellow clearly has better practical usage than BOTCP (MD). MEGA PROTAC provided better ranking accuracy for almost every threshold. Particularly, MEGA PROTAC hit 100% accuracy for the threshold of 20, while BOTCP (MD) provided around 80% accuracy for the same threshold. Consequently, MEGA PROTAC clearly has potential for practical usage, while it has comparative performance against BOTCP (MD).

1.7.4 Limitations of BOTCP

BOTCP has been suffering from limited performance, especially in the pre-refinement step. While BOTCP is not an open-source algorithm, its scoring functions, such as PPI and Constraint fitness, may not comprehensively describe ternary structures, which could contribute to its subpar performance. Another possible explanation for BOTPC's limited performance is using TCP-AIR energy in cluster filtering. Energy-based filtration cannot be the optimal choice, as the energy scoring mechanism in the FRODOCK and RosettaDock-based pipelines requires additional ranking methods. To enhance performance in PROTAC screening by overcoming these limitations, MEGA PROATC was developed and has been evaluated alongside BOTCP as a cutting-edge therapy in a comparative analysis.

BOTCP has exhibited subpar quality and restricted usefulness compared to MEGA PROTAC. BOTCP used 7D the relative rotation and translation (RRT) representation for PPI and constraint scores in the bayesian optimization (BO) loop, however these can limit the performance of BOTCP because of insufficient data to

optimise their scoring approach. On the other hand, the neural network model's excessive complexity may result in overfitting or underfitting, leading to a loss of its overall function because of limited data. The scoring function utilised by BOTCP assesses the efficacy of the PROTAC molecule in binding to PPC and its ability to facilitate the closeness of the proteins. This function may have imperfections and may overlook certain crucial aspects. Another limitation of BOTCP comes from TCP-AIR energy filtration. Since TCP-AIR filtering only keeps the top 10% of proteins, each cluster keeps one for further BOTCP protocol. However, our research shows that energy-based filtration is not the most effective alternative. The BOTCP, particularly the molecular dynamic simulation application, is a method that requires significant processing resources and a lot of time to improve the low performance of pre-refinement. Inadequate computational resources can hinder the model's ability to thoroughly explore the whole search space and identify the optimal answer. Consequently, BOTCP is a computationally expensive and time-consuming method.

MEGA PROTAC employs six distinct quality and filtration methodologies to analyse structures from diverse perspectives, with the goal of addressing the shortcomings of BOTCP. These approaches have already been optimised and validated in their original papers. Therefore, unlike BOTCP, the scarcity of ternary structures is no longer a concern for MEGA PROTAC, as BOTCP relies on a restricted amount of data to train its models and optimise PPI and Constraint Fitness functions. MEGA PROTAC benefits from rank aggregation's robustness and high-performance characteristics, which improves its performance when rating PPCs. One notable advantage of MEGA PROTAC is the use of SASA to estimate protein proximity, particularly in locations where PROTAC may be accommodated. Furthermore, the use of VoroMQA and SASA in rank aggregation resulted in increased robustness and higher performance, as previously stated.

1.8 Examining Ternary Structure Prediction for Methods via Visual Analysis for BOTCP and MEGA PROTAC

The first and most difficult issue in determining the conformation of ternary structures for PROTAC is the identification of suitable protein-protein complexes. It is both challenging and crucial for three primary reasons: (i) Protein architectures within PROTAC-induced ternary structures differ from ordinary protein-protein complexes, which need a greater spacing between proteins compared to the usual arrangement. Conventional PPI methods tend to prioritise stable protein complexes with larger interaction interfaces. However, an increased protein interface leads to a proportionally reduced binding site for PROTAC; therefore, their performance is limited when constructing PROTAC-mediated ternary structures. In order to overcome the problem, although existing models use multiple methods to increase performance in the identification of ternary structures by increasing the robustness of their methods, they have been suffering limited performance. (ii) The second problem arises from the disparity in mass among the three components. Given the substantial size difference between proteins and PROTACs, it is necessary to prioritise the optimisation of protein complex structures in order to get higher-quality structures. After finding an acceptable protein complex structure, although the initial pose of PROTAC may be incorrect, it

can be enhanced by utilising local redocking approaches that offer a great degree of flexibility. Otherwise, optimising a protein or both proteins using computational methods entails beginning anew. (iii) Once protein complexes are accurately predicted, they serve as an ideal "lock" for the PROTAC, which acts as the corresponding "key". Hence, identifying protein complexes, even without the information of a warhead and anchor on proteins, will greatly enhance the efficiency of ternary structure creation. Consequently, prior research mostly concentrated on evaluating the efficacy of their techniques in identifying the optimal protein complex because of these reasons.

In order to mitigate possible bias, our primary focus has been on evaluating the performance of protein complexes using approaches similar to those employed in earlier investigations, such as DockQ score. However, to examine the correlation between the quality of protein complex structures and the success of PROTAC posture, three structures have been randomly selected, namely 5T35-HE, 7JTP-LA, and 7KHH-CD, as a case study to compare MEGA PROTAC with BOTCP (Figure 2).

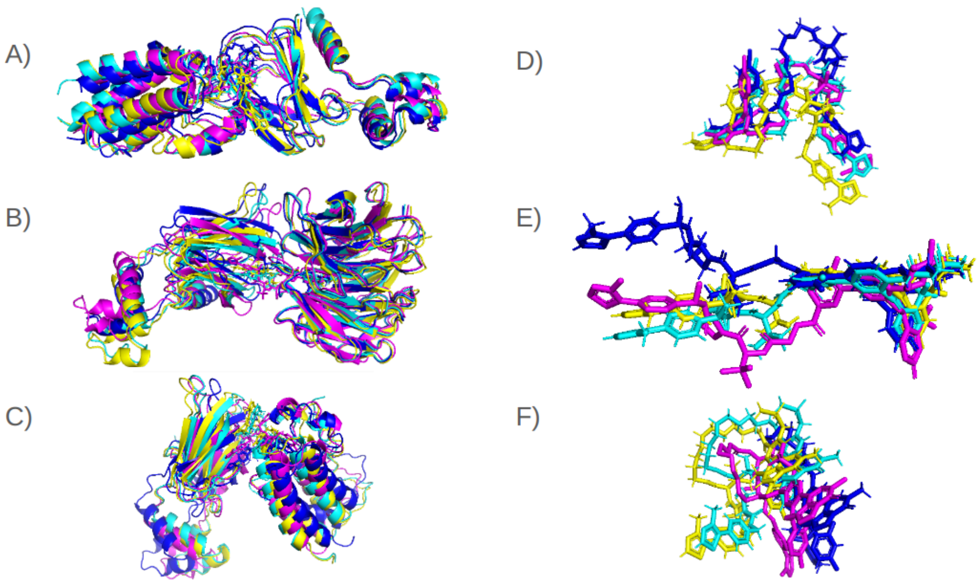

Fig. 2 The figure illustrates ternary structure models by using Pymol. A indicates the ternary structure of 5T35-HE, while D displays the poses of PROTAC for 5T35-HE. B displays the 7JTP-LA ternary structure, while E indicates the PROTAC pose for 7JTP-LA. The third ternary structure model and PROTAC pose for 7KHH-CD have been demonstrated in C and F, respectively. Four distinct colours represent different aspects of the true ternary structure: cyan for true ternary structure, yellow for MEGA PROTAC, blue for BOTCP-pre-refinement, and magenta for BOTCP-MD.

The 5T35-HE ternary structure models and ligand poses are demonstrated in Figures 2 A and D. Most of the beta sheets on 5T35-HE have been correctly predicted by each method, including MEGA PROTAC and BOTCP methods. Also, the alpha helices on the right and left side of Figure 2 A and D clearly demonstrated

that MEGA PROTAC (yellow) and BOTCP-MD (magenta) models highly match with ground truth (cyan) structure. At the same time, BOTCP pre-refinement has mismatched these alpha helices. The same trend has been observed on the PROTAC pose (Figure 2 D): the PROTAC pose performance of MEGA PROTAC (yellow) and BOTCP-MD (magenta) performance is higher than BOTCP (pre-refinement). Consequently, MEGA PROTAC outperformed BOTCP (pre-refinement), while it is competitive against the molecular dynamic simulation application of BOTCP.

Figure 2 B and E illustrate the case study analysis of the ternary structure and PROTAC orientations of 7JTP-LA. The right side of the ternary structure (Figure 2 B) has been accurately modelled by each of the methods. However, on the left side of the ternary structure, MEGA PROTAC (in yellow) provided the best model to describe ground truth (in cyan). Both the pre-refinement version of BOTCP (in blue) and the MD of BOTCP (in magenta) exhibit significant mismatches in the beta sheets on the left side, which are close to the PROTAC molecule and alpha helices at the end of the left side of the ternary structure, respectively. Regarding the PROTAC postures (Figure 2 E), the MEGA PROTAC (yellow) exhibited the most accurate motif with the actual structure (cyan) through machining. The molecular dynamic simulation application of BOTCP (magenta) and pre-refinement BOTCP (blue) hardly matches the motif with the ground truth (cyan).

Table 5 The table displays the first cycle RMSD values for three case study protein structures, including 5T35-HE, 7JTP-LA and 7KHH-CD. The first cycle RMSD for the entire ternary structure was calculated from the align command in Pymol. Three models have been employed to compare the performance of ternary structures for MEGA PROTAC and BOTCP techniques.

**Ternary Structures**	**Methods**		
	**MEGA PROTAC**	**BOTCP (pre-refinement)**	**BOTCP (MD)**
5T35-HE	6.82	6.97	**6.61**
7JTP-LA	**2.23**	3.61	2.96
7KHH-CD	**1.7**	6.17	3.99

The last case study about 7KHH-CD has been examined and is depicted in Figures 2 C and F. The construction of the 7KHH-CD ternary structure is highly difficult due to the intricate bending of each protein, which may potentially result in the formation of spurious binding sites. Such a curvature can enhance the surface area between proteins, leading to increased stability. The increased stability of proteins can be misleading when determining the right ternary structure using certain approaches. Therefore, 7KHH-CD was used as a challenging case study example to assess the performance of the methods. While BOTCP (MD) (magenta) accurately matched the alpha helices on both the right and left sides of the ternary structure, BOTCP (pre-refinement) failed to match these helices and also mismatched the beta sheets on the left side of the PROTAC poses Figures 2. However, MEGA PROTAC demonstrated superior performance by accurately predicting most beta sheets and alpha helices. Regarding the performance of techniques in terms of PROTAC posture (Figure 2 F), both BOTCP approaches (blue and magenta) exhibited poses that were deemed undesirable since they did not closely resemble the true theme (cyan). However, MEGA

PROTAC (yellow) nearly discovered the ideal pattern for PROTAC, with only a minor translational mistake. The examples also demonstrated that MEGA PROTAC performed superior in the 7KHH-CD case study against BOTCP results.

To assess the overall efficacy of the methods in constructing ternary structures, RMSD values for the ternary structures were obtained using the Pymol align command, so Table 5 displays the RMSDs for the first cycle when the align command is used in Pymol. In the initial cycle, the lowest number of atoms is often excluded from determining the RMSDs for the structures; therefore, this calculation is performed using the majority of the atoms in the structure. Therefore, the first cycle RMSD was utilized to evaluate and compare the efficiency of ternary structure assembly. Based on the RMSD values in Table 5, it can be observed that MEGA PROTAC had the lowest RMSD, except 5T35-HE, where BOTCP (MD) has the lowest RMSD. Consequently, MEGA PROTAC provided promising performance because of its well-designed filtration integrated with rank aggregation. MEGA PROTAC not only outperformed BOTCP (pre-refinement) but is also competitive or better against the time-consuming and computationally intense molecular dynamic simulation application of BOTCP.

1.8.1 The performance of MEGA PROTAC assessment via Visual Analysis

Utilising tools such as PyMOL [30] for visual analysis is essential for comprehending the structural dynamics and interactions within protein-protein complexes. Visualising and analysing protein complexes in three dimensions offers vital insights into their behaviour, structural changes, and binding sites. PyMOL is a popular software for visualising molecules, which has enhanced capabilities that make it easier to study and understand complicated biomolecular structures. This study emphasises the significance of visual analysis in evaluating MEGA PROTAC's effectiveness, a tool for predicting protein-protein interactions. Using PyMOL, the evolutionary changes in protein complexes during the grid search application are illustrated. This allows us to get insights into the structural modifications and interaction patterns caused by using MEGA PROTAC. This visual analysis aims to thoroughly comprehend the dynamic behaviour of protein complexes and the effectiveness of MEGA PROTAC in forecasting protein-protein interactions. As a result, 22 test cases have been demonstrated in Figures 3 and 4.

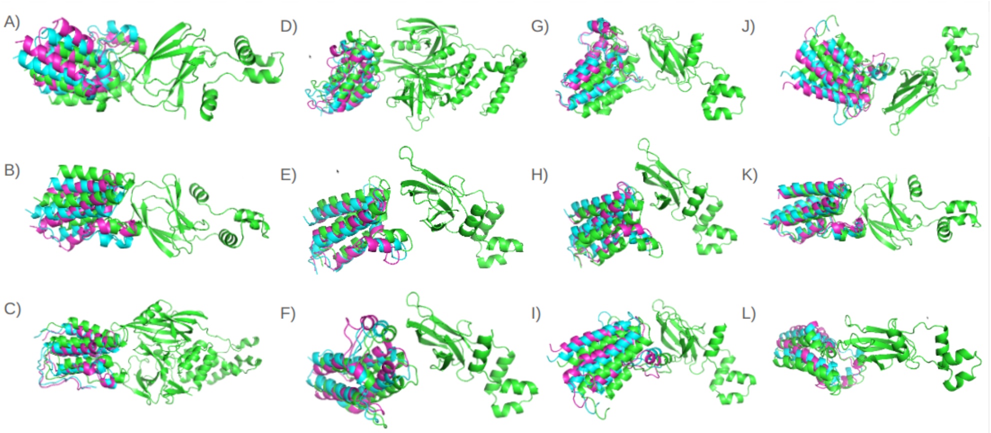

Fig. 3 The figure virtually demonstrates how ligand-protein structures changed from MEGADOCK pre-grid refinement candidate PPC to the highest DockQ score structure. The green structure demonstrates the MEGADOCK pre-grid refinement candidate PPC, cyan represents a translated protein, and the magenta shows the rotated structure as a final pose having the highest DockQ score. In order, 5T35-DA, 5T35-HE, 6BN7-BC, 6BOY-BC, 6HAX-BA, 6HAX-FE, 6HAY-BA, 6HAY-FE, 6HR2-BA, 6HR2-FE, 6SIS-DA, 6SIS-HE were represented in A, B, C, D, E, F, G, H, I, J, K and L.

Figure 3 displays a sequence of twelve PPCs, namely 5T35-DA, 5T35-HE, 6BN7-BC, 6BOY-BC, 6HAX-BA, 6HAX-FE, 6HAY-BA, 6HAY-FE, 6HR2-BA, 6HR2-FE, 6SIS-DA, and 6SIS-HE. The alpha helices depicted in Figure 3 A indicate that changes in the location and rotation of the ligand-protein complex lead to an improvement in the DockQ score. This is evident from the distinct separation observed in the position of the alpha helices. Figure 3 A unequivocally shows that changing the location and rotation significantly increases the DockQ score from 0.546 to 0.778, as indicated in Table 4. The remaining portion of the illustration in Figure 3 further confirms that both translational and rotational modifications play a role in improving the DockQ score. Figure 3 demonstrates the potential of the grid search method used by MEGA PROTAC to develop specialised molecular docking software for PROTAC screening.

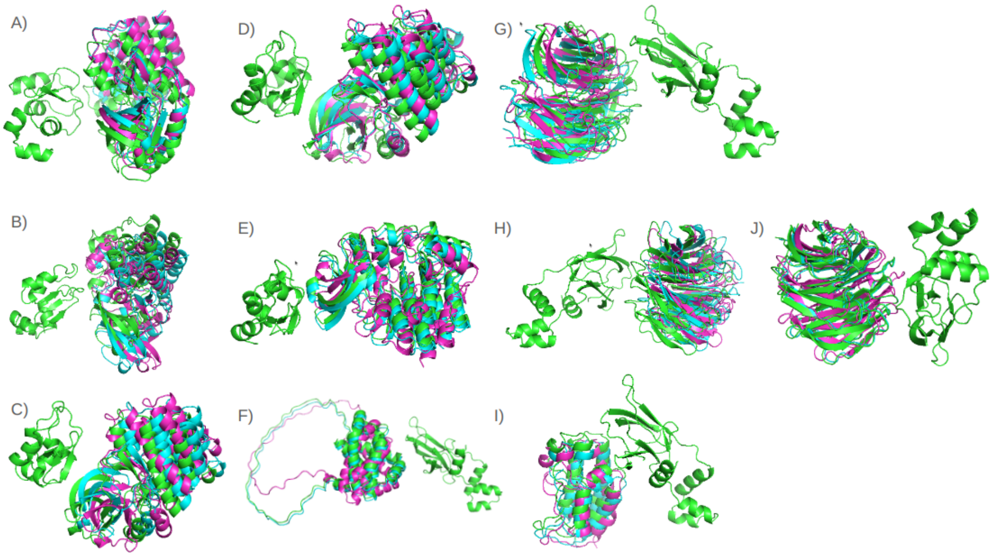

Fig. 4 The figure virtually demonstrates how ligand structures changed from the MEGADOCK pre-grid refinement candidate PPC to the structure with the highest DockQ score. The green structure demonstrates the MEGADOCK pre-grid refinement candidate PPC, cyan represents a translated protein, and magenta shows the rotated structure as a final pose with the highest DockQ score. In order, 6W7O-CA, 6W7O-DB, 6W8I-DA, 6W8I-EB, 6W8I-FC, 6ZHC-AD, 7JTO-LB, 7JTP-LA, 7KHH-CD, 7Q2J-CD were represented in A, B, C, D, E, F, G, H, I, and J.

Figure 4 supports the results discussed in Figure 3. Figure 4 F shows a strange output. A chain far from the main protein body is in the wrong position for 6ZHC-AD. This example clearly demonstrates the limitation of grid docking, such as MEGA PROTAC. Although the chain is in the wrong position, the DockQ score of 0.417 for 6ZHC-AD demonstrates that MEGA PROTAC found most of the structure's backbone.

The data in Table 5 indicates that the MEGA PROTAC technique outperforms the BOTCP methods (both pre-refinement and MD) to achieve lower RMSD values for the ternary protein structures examined. Specifically, MEGA PROTAC yields the lowest RMSD values for 7JTP-LA (2.23) and 7KHH-CD (1.7), demonstrating better structural alignment and stability than BOTCP techniques. Even in the case of 5T35-HE, while BOTCP (MD) shows a slightly lower RMSD (6.61) than MEGA PROTAC (6.82), the differences are marginal. Overall, the consistently lower RMSD values for MEGA PROTAC across multiple structures suggest that this method provides a more reliable and precise approach for modelling ternary structures.

1.9 MEGADOCK Parameters

MEGADOCK, the software for molecular docking, utilises a meticulously calibrated set of criteria to enhance the precision and effectiveness of forecasting protein-protein interactions (Table 6). The value of the output_number option is set to 5000, which

signifies that the software will produce and analyse a maximum of 5000 potential docking poses. The voxel size is set to 3, determining the spatial grid resolution utilised for mapping protein surfaces. The degree parameter, set to 12, determines the level of detail in the angular sampling during the docking procedure. MEGADOCK does rotate sampling at 54000 distinct orientations, hence increasing the comprehensiveness of the search for ideal docking configurations. The rPSC receptor core penalty and the rPSC ligand core penalty parameters, with values of -1000 and 10, respectively, are energy-related factors that impact the evaluation and ordering of docking postures. Ultimately, the function parameter is assigned a value of 1, indicating the specific scoring system or weight utilised to assess each docking position's excellence. Combining these factors allows MEGADOCK to conduct thorough and accurate docking simulations, making identifying biologically significant protein-protein interactions easier.

Table 6 This table enumerates the essential parameters employed by MEGADOCK, a sophisticated molecular docking program developed to enhance the accuracy and efficiency of predicting protein-protein interactions. These parameters are critical for the program's performance, influencing the docking accuracy and computational efficiency.

**Parameter**	**Summary**
-o filename	set the output filename (default to "R–L.out")
-O	output docking detail files
-N integer	set the number of output predictions (default to 2000)
-t integer	set the number of predictions per each rotation (default to 1)
-F integer	set the number of FFT point (default to none)
-v float	set the voxel size (default to 1.2)
-D	set the 6 deg. (54000 angles) of rotational sampling<br>(default to none, 15 deg. (3600 angles) of rotational sampling)
-r integer	set the number of rotational sampling angles<br>(54000: 54000 angles, 1: 1 angles, 24: 24 angles, default to 3600 angles)
-e float	set the electrostatics term ratio (default to 1.0)
-d float	set the hydrophobic term ratio (default to 1.0)
-a float	set the rPSC receptor core penalty (default to -45.0)
-b float	set the rPSC ligand core penalty (default to 1.0)
-f 1/2/3	set function (default to 3)

By optimising the parameters of MEGA PROTAC, its sampling performance can be greatly improved. Researchers can accomplish a more accurate and efficient exploration of the protein-protein interaction space by adjusting parameters such as output number, grid size, sampling degree, the rPSC receptor core penalty, and the rPSC ligand core penalty. Increasing the output number enables the MEGA PROTAC to generate more alternative docking postures, enhancing the probability of detecting the best protein-protein complexes for PROTAC. Modifying the grid size and sampling degree parameters can enhance the spatial and angular resolution, resulting in more precise docking of protein-protein complexes. In addition, adjusting the energy-related parameters, such as the rPSC receptor core penalty and the rPSC ligand core penalty, can improve the accuracy and order of docking poses, thus improving the overall quality of the results.

Another efficient approach to enhance sampling performance is to run MEGADOCK numerous times with different parameters. By conducting several iterations using various configurations, researchers can explore a wider range of the parameter space, thereby enhancing the likelihood of locating the optimal docking postures. This approach enables a thorough investigation of the potential interactions, as different parameter sets might encompass diverse features of the docking scene. Every execution can be customised to highlight various features of the docking process, such as prioritising particular angular resolutions or energy thresholds. By combining the outcomes of these several iterations, scientists can acquire a more resilient and dependable collection of docking forecasts, ultimately enhancing the overall efficiency and precision of the sampling procedure.

Adjusting the parameters of MEGA PROTAC through fine-tuning and running MEGADOCK numerous times with different setups is advantageous to boost sampling performance. Implementing these methodologies can enhance the accuracy and comprehensiveness of identifying protein-protein interactions, a critical aspect for furthering research and development in computational biology and drug discovery.

References

[1] José Ignacio Garzon, José Ramón Lopéz-Blanco, Carles Pons, Julio Kovacs, Ruben Abagyan, Juan Fernandez-Recio, and Pablo Chacon. Frodock: a new approach for fast rotational protein–protein docking. *Bioinformatics*, 25(19):2544–2551, 2009.

[2] Sergey Lyskov and Jeffrey J Gray. The rosettadock server for local protein–protein docking. *Nucleic acids research*, 36(suppl_2):W233–W238, 2008.

[3] Gaoqi Weng, Dan Li, Yu Kang, and Tingjun Hou. Integrative modeling of protac-mediated ternary complexes. *Journal of Medicinal Chemistry*, 64(21):16271–16281, 2021.

[4] Daniel Zaidman, Jaime Prilusky, and Nir London. Prosettac: Rosetta based modeling of protac mediated ternary complexes. *Journal of chemical information and modeling*, 60(10):4894–4903, 2020.

[5] Diogo Santos-Martins, Stefano Forli, Maria João Ramos, and Arthur J Olson. Autodock4zn: an improved autodock force field for small-molecule docking to zinc metalloproteins. *Journal of chemical information and modeling*, 54(8):2371–2379, 2014.

[6] Piyush Agrawal, Harinder Singh, Hemant Kumar Srivastava, Sandeep Singh, Gaurav Kishore, and Gajendra PS Raghava. Benchmarking of different molecular docking methods for protein-peptide docking. *BMC bioinformatics*, 19:105–124, 2019.

[7] Thomas Eckart Exner, Oliver Korb, and Tim Ten Brink. New and improved features of the docking software plants. *Chemistry Central Journal*, 3(Suppl 1):P16, 2009.

[8] Jinsol Yang, Minkyung Baek, and Chaok Seok. Galaxydock3: Protein–ligand docking that considers the full ligand conformational flexibility. *Journal of Computational Chemistry*, 40(31):2739–2748, 2019.

[9] Nicholas A Marze, Shourya S Roy Burman, William Sheffler, and Jeffrey J Gray. Efficient flexible backbone protein–protein docking for challenging targets. *Bioinformatics*, 34(20):3461–3469, 2018.

[10] Daniel Varela, Vera Karlin, and Ingemar André. A memetic algorithm enables efficient local and global all-atom protein-protein docking with backbone and side-chain flexibility. *Structure*, 30(11):1550–1558, 2022.

[11] Sadettin Y Ugurlu, David McDonald, Huangshu Lei, Alan M Jones, Shu Li, Henry Y Tong, Mark S Butler, and Shan He. Cobdock: an accurate and practical machine learning-based consensus blind docking method. *Journal of Cheminformatics*, 16(1):5, 2024.

[12] Masahito Ohue, Takehiro Shimoda, Shuji Suzuki, Yuri Matsuzaki, Takashi Ishida, and Yutaka Akiyama. Megadock 4.0: an ultra–high-performance protein–protein docking software for heterogeneous supercomputers. *Bioinformatics*, 30(22):3281–3283, 2014.

[13] Takehiro Shimoda, Takashi Ishida, Shuji Suzuki, Masahito Ohue, and Yutaka Akiyama. Megadock-gpu: acceleration of protein-protein docking calculation on gpus. In *Proceedings of the International Conference on Bioinformatics, Computational Biology and Biomedical Informatics*, pages 883–889, 2013.

[14] Rong Chen, Li Li, and Zhiping Weng. Zdock: an initial-stage protein-docking algorithm. *Proteins: Structure, Function, and Bioinformatics*, 52(1):80–87, 2003.

[15] Brian Jiménez-García, Jorge Roel-Touris, Miguel Romero-Durana, Miquel Vidal, Daniel Jiménez-González, and Juan Fernández-Recio. Lightdock: a new multi-scale approach to protein–protein docking. *Bioinformatics*, 34(1):49–55, 2018.

[16] Brian G Pierce, Kevin Wiehe, Howook Hwang, Bong-Hyun Kim, Thom Vreven, and Zhiping Weng. Zdock server: interactive docking prediction of protein–protein complexes and symmetric multimers. *Bioinformatics*, 30(12):1771–1773, 2014.

[17] Richard J Gowers, Max Linke, Jonathan Barnoud, Tyler John Edward Reddy, Manuel N Melo, Sean L Seyler, Jan Domanski, David L Dotson, Sébastien Buchoux, Ian M Kenney, et al. Mdanalysis: a python package for the rapid analysis of molecular dynamics simulations. Technical report, Los Alamos National Laboratory (LANL), Los Alamos, NM (United States), 2019.

[18] Virginie Y Martiny, Pablo Carbonell, David Lagorce, Bruno O Villoutreix, Gautier Moroy, and Maria A Miteva. In silico mechanistic profiling to probe small molecule binding to sulfotransferases. *PLoS One*, 8(9):e73587, 2013.

[19] Simon Mitternacht. Freesasa: An open source c library for solvent accessible surface area calculations. *F1000Research*, 5, 2016.

[20] Noel M O'Boyle, Michael Banck, Craig A James, Chris Morley, Tim Vandermeersch, and Geoffrey R Hutchison. Open babel: An open chemical toolbox. *Journal of cheminformatics*, 3:1–14, 2011.

[21] Arjun Rao, Tin M Tunjic, Michael Brunsteiner, Michael Müller, Hosein Fooladi, Chiara Gasbarri, and Noah Weber. Bayesian optimization for ternary complex prediction (botcp). *Artificial Intelligence in the Life Sciences*, 3:100072, 2023.

[22] Ozlem Keskin, Nurcan Tuncbag, and Attila Gursoy. Predicting protein–protein interactions from the molecular to the proteome level. *Chemical reviews*,

116(8):4884–4909, 2016.

[23] Ankit A Roy, Abhilesh S Dhawanjewar, Parichit Sharma, Gulzar Singh, and MS Madhusudhan. Protein interaction z score assessment (pizsa): an empirical scoring scheme for evaluation of protein–protein interactions. *Nucleic acids research*, 47(W1):W331–W337, 2019.

[24] Marco Cinelli, Miłosz Kadziński, Michael Gonzalez, and Roman Słowiński. How to support the application of multiple criteria decision analysis? let us start with a comprehensive taxonomy. *Omega*, 96:102261, 2020.

[25] Shili Lin. Rank aggregation methods. *Wiley Interdisciplinary Reviews: Computational Statistics*, 2(5):555–570, 2010.

[26] Sankar Basu and Björn Wallner. Dockq: a quality measure for protein-protein docking models. *PloS one*, 11(8):e0161879, 2016.

[27] Marc F Lensink, Nurul Nadzirin, Sameer Velankar, and Shoshana J Wodak. Modeling protein-protein, protein-peptide, and protein-oligosaccharide complexes: Capri 7th edition. *Proteins: Structure, Function, and Bioinformatics*, 88(8):916–938, 2020.

[28] Hernan Alonso, Andrey A Bliznyuk, and Jill E Gready. Combining docking and molecular dynamic simulations in drug design. *Medicinal research reviews*, 26(5):531–568, 2006.

[29] Lucianna HS Santos, Rafaela S Ferreira, and Ernesto R Caffarena. Integrating molecular docking and molecular dynamics simulations. *Docking screens for drug discovery*, pages 13–34, 2019.

[30] Shuguang Yuan, HC Stephen Chan, and Zhenquan Hu. Using pymol as a platform for computational drug design. *Wiley Interdisciplinary Reviews: Computational Molecular Science*, 7(2):e1298, 2017.
